# The static and dynamic structural heterogeneities of B-DNA: extending Calladine–Dickerson rules

**DOI:** 10.1093/nar/gkz905

**Published:** 2019-10-18

**Authors:** Pablo D Dans, Alexandra Balaceanu, Marco Pasi, Alessandro S Patelli, Daiva Petkevičiūtė, Jürgen Walther, Adam Hospital, Genís Bayarri, Richard Lavery, John H Maddocks, Modesto Orozco

**Affiliations:** 1 Institute for Research in Biomedicine (IRB Barcelona). The Barcelona Institute of Science and Technology. Baldiri Reixac 10–12, 08028 Barcelona, Spain; 2 Department of Biological Sciences, University of the Republic (UdelaR), CENUR Gral. Rivera 1350, 50000 Salto, Uruguay; 3 LBPA, École normale supérieure Paris-Saclay, 61 Av. du Pdt Wilson, Cachan 94235, France; 4 Bases Moléculaires et Structurales des Systèmes Infectieux, Univ. Lyon I/CNRS UMR 5086, IBCP, 7 Passage du Vercors, Lyon 69367, France; 5 Institute of Mathematics, Swiss Federal Institute of Technology (EPFL), CH-1015 Lausanne, Switzerland; 6 Faculty of Mathematics and Natural Sciences, Kaunas University of Technology, Studentų g. 50, 51368 Kaunas, Lithuania; 7 Department of Biochemistry and Molecular Biology. University of Barcelona, 08028 Barcelona, Spain

## Abstract

We present a multi-laboratory effort to describe the structural and dynamical properties of duplex B-DNA under physiological conditions. By processing a large amount of atomistic molecular dynamics simulations, we determine the sequence-dependent structural properties of DNA as expressed in the equilibrium distribution of its stochastic dynamics. Our analysis includes a study of first and second moments of the equilibrium distribution, which can be accurately captured by a harmonic model, but with nonlocal sequence-dependence. We characterize the sequence-dependent choreography of backbone and base movements modulating the non-Gaussian or anharmonic effects manifested in the higher moments of the dynamics of the duplex when sampling the equilibrium distribution. Contrary to prior assumptions, such anharmonic deformations are not rare in DNA and can play a significant role in determining DNA conformation within complexes. Polymorphisms in helical geometries are particularly prevalent for certain tetranucleotide sequence contexts and are always coupled to a complex network of coordinated changes in the backbone. The analysis of our simulations, which contain instances of all tetranucleotide sequences, allow us to extend Calladine–Dickerson rules used for decades to interpret the average geometry of DNA, leading to a set of rules with quantitative predictive power that encompass nonlocal sequence-dependence and anharmonic fluctuations.

## INTRODUCTION

DNA is a flexible and structurally polymorphic polymer whose overall equilibrium geometry strongly depends on its sequence, the solvent environment, and the presence of ligands (1,2). Conformational changes in DNA are mediated by a complex choreography of backbone rearrangements such as the BI/BII transition (3,4), the low-twist/high-twist equilibrium (5,6), or concerted α/γ rotations (7–9). Such backbone rearrangements lead to local and global changes in the helix geometry (9,10) impacting on the ability of the DNA to recognize ligands (11), and consequently on its functionality.

Binding-induced conformational changes in DNA are required for function and are expected to follow the sequence-dependent intrinsic deformation modes of DNA, *i.e*. are implicitly coded in the spontaneous deformability of isolated DNA. This suggests that evolution has refined DNA sequence not only to maximize ligand-DNA interactions but also to reduce the energetic cost of moving from a canonical to a bioactive conformation (11,12). This leads the notion of ‘indirect readout’, which suggests that the ability of the DNA to adopt the ‘bioactive’ conformation plays a major role in determining the target sequences of a given DNA ligand. Understanding the sequence-dependent physical properties of DNA then becomes crucial to rationalizing how ligands and, most notably, proteins, recognize and modulate DNA activity, i.e. the structural basis of gene regulation.

Understanding the sequence-dependent physical properties of DNA has been traditionally hampered by the lack of experimental data. Using simple steric considerations and geometric constraints Calladine developed in 1982 a set of principles to describe the mechanics of DNA (13), which have been used for decades to gain some qualitative insight into the sequence-dependence of expected, or average, local helical geometry. In their original version, those principles suggested that clashes between bases are avoided by a combination of a concerted change in twist, roll, and slide, as the basepair propeller increases to improve stacking (13). One year later, Dickerson formulated a simple numerical algorithm allowing for a quantification of Calladine's principles, coining the procedure as the ‘Calladine's Rules’ (14,15), which explains qualitatively the local variation (at the basepair level) in twist, roll, propeller twist and the torsion angle δ for a few B-DNA sequences which were previously determined experimentally (14). Unfortunately, the extension and predictive power of these rules, even in the most recent versions, is limited (1,16). Attempts to gain more quantitative information were based on the analysis of the variability in local helical parameters in structural databases (17,18), but to date (data accessed on the 19 March 2019), isolated B-DNA structures in the Nucleic acid Data Bank (NDB) allowed us to obtain flexibility data for only 5 of the 136 distinct tetranucleotides (only AATT, CGCG, CGAA, GCGA and ATTC are represented more than 500 times from 10 134 tetranucleotide analyzed belonging to 727 PDB structures). Even when the database is extended by including protein–DNA complexes (155 316 tetranucleotides belonging to 3434 PDB structures), the sampling is not dense enough to describe sequence-dependent DNA flexibility at the tetranucleotide level (24 out of the 136 tetranucleotides are still represented less than 500 times). In this context, atomistic molecular dynamics (MD) simulations are the only alternative to obtain robust and transferable parameters (10,19,20).

The first requirement for deriving physical descriptors of DNA from MD simulations is the availability of extended simulations for a library of sequence fragments containing all distinct tetranucleotides. This requires a significant computational effort which has encouraged joint projects such as the Ascona B-DNA Consortium (ABC, https://bisi.ibcp.fr/ABC), which have been instrumental, not only in describing physical properties of DNA but also in refining simulation protocols (10,21–23). The second major requirement is the availability of accurate force fields, such as the recently developed PARMBSC1 (24), which has been shown to represent DNA with a quality indistinguishable from experimental measurements (25). Thanks to the coordinated effort of several ABC groups, a series of microsecond-scale simulations on a library of DNA duplexes covering all of the 136 distinct tetranucleotides have been performed, and with a number of different simulation conditions, e.g. using PARMBSC0 (26) or PARMBSC1, different counter ions, etc. Consequently, there is a minimum of six total simulations of each independent tetranucleotide. The analysis of this large ensemble of data allows us to not only decipher the rules defining the sequence-dependent equilibrium geometry of B-DNA, but also those determining coordinated backbone conformational changes, and the correlations between various helical deformations. A new and comprehensive extension of Calladine–Dickerson rules emerges from the analysis of these simulations, including the first predictions of anharmonicity based on sequence context.

## MATERIALS AND METHODS

### The choice of sequences

The new ABC sequence library was designed to optimize the number of relatively short oligomers needed to include one copy of each of the distinct 136 tetranucleotides. Applying an adapted version of the Orenstein and Shamir algorithm (27–29), we generated 13 oligomers, each containing 18 bp (including GC terminals on each end), covering the complete tetranucleotide space (see Supplementary Table S1 for a list of the designed sequences), and 117 (of the 2080 possible) distinct hexanucleotide sequences. The smaller number of oligomers with respect to previous training libraries (6,10) made it more practical to obtain multi-microsecond trajectories under several simulation conditions (e.g. using both the PARMBSC1 (24) and PARMBSC0 (30) force fields, labelled miniABC_BSC1_ and miniABC_BSC0_ respectively), and by changing the ionic environment (from KCl to NaCl, labelled miniABC_BSC1_-K and miniABC_BSC1_-Na respectively). Comparison of results obtained with this library of sequences (miniABC) with respect to the standard ABC-set (μABC (10)) allowed us to check for the robustness of our conclusions as a function of the duplexes from which the tetranucleotide parameters were derived.

### System preparation and MD simulations

All oligonucleotides were constructed with the *leap* program of AMBERTOOLS 15 (31) and simulated using the *pmemd.cuda* code (32) from AMBER14 (31), following the standard ABC protocol (10). Canonical duplexes were generated using Arnott B-DNA fiber parameters (33), and solvated by a truncated octahedral box of SPC/E (34) water molecules with a minimum distance of 10 Å between DNA and the closest face of the box. Systems were neutralized with K^+^ or Na^+^ ions adding additional 150 mM of K^+^Cl^−^ (or Na^+^Cl^−^). PARMBSC0 (30) and PARMBSC1 (24) force fields were used to describe DNA, while Dang's parameters were used for ions (35). Systems were optimized and equilibrated as described elsewhere (10), and simulated for 1 μs in the NPT ensemble, using Particle-Mesh Ewald corrections (36) and periodic boundary conditions. SHAKE was used to constrain bonds involving hydrogen (37), allowing 2 fs integration step. Typically, analyses presented here correspond to the second part of the trajectory (last 500 ns). Trajectories are accessible at the BIGNASim server: https://mmb.irbbarcelona.org/BIGNASim/ (38).

### Global analysis

Trajectories were processed with the *cpptraj* (39) module of the AMBERTOOLS 15 package (31), and the NAFlex server (40) for standard analysis. DNA helical parameters and backbone torsion angles were measured and analysed with the CURVES+ and CANAL programs (41), following the standard ABC conventions (10). Duplexes were named following the Watson strand. The letters R, Y and X stand for a purine, a pyrimidine, or any base respectively; base pairs flanking a dinucleotide step were denoted using two dots to represent the central step (e.g. R..Y), and one dot when trinucleotides are considered (e.g. R.Y), while X:X and XX represent a basepair and a basepair step, respectively. Bayesian Information Criterion (or BIC) (42,43) was used to quantify the normal or binormal (i.e. a mixture of two normal functions) nature of the distributions of the helical parameters (see Supplementary Methods). An extension of Helguerro's theorem (44,45) was used to distinguish those binormal distributions where the two Gaussians are very close (unimodal distributions) from those where they are significantly separated (bimodal distributions). The similarity between first and second moments (i.e. average and covariance) of the helical parameter distributions for different simulation libraries was evaluated using the Kullback-Leibler (KL) divergence. More specifically sequence-dependent Gaussian coarse grain cgDNA (46–48) model parameters were computed from each of the four MD training libraries used in this work (i.e. μABC_BSC0_-K, miniABC_BSC0_-K, miniABC_BSC1_-K, miniABC_BSC1_-Na) in order to be able to generate associated predictions of first and second moments of the helical parameters for fragments of arbitrary sequence. In particular this allowed us to compare PARMBSC0 simulations of the μABC library with the PARMBSC0 simulations of the miniABC library, even though the two libraries have different sequence fragments. See the Supporting Methods for specific details on BIC, Bayes Factors, Helguerro's theorem, KL divergence between configuration distributions, and DNA Persistence Length (PL) calculations.

### Correlations between helical structural substates

For each tetranucleotide we calculated the correlation between the backbone state at the central step (inter-basepair *i*) and the helical parameters at two consecutive levels around the central dinucleotide (*i −* 1, *i* and *i* + 1). The substates of the torsion angles of the backbone were categorized following the standard definition: *gauche* positive (*g*+) = 60 ± 40°; *trans* (*t*) = 180 ± 40° and gauche negative (*g*−) = 300 ± 40°. For the correlations with BI/BII, we assigned to the backbone one of two possible discrete values, either BI or BII, according to the sub-state of the ζ torsion (*g*− or *t* respectively) at the central bps junction. All frames where the ζ torsion did not fall inside the ranges defined by *g*− and *t* were not considered in the analysis. This leads to a strong reduction of the noise that comes from specific tetranucleotides, when trying to find patterns by grouping them (e.g. the ‘noise’ arising from the individual behavior of the GAGA, GGGG and AAGA tetranucleotides when considering the RRRR family). The point-biserial (49) correlation coefficient, mathematically equivalent to the Pearson correlation (50), was used as a measure of the correlation between these discrete substates of the backbone and the continuous values of the inter-basepair helical parameters. The obtained correlation values were divided in five categories: (i) ≥−0.6, strong negative correlation; (ii) <−0.6 and ≥−0.4, mild negative correlation; (iii) >−0.4 and <0.4, no correlation; (iv) ≥0.4 and <0.6, mild positive correlation and finally (v) ≥0.6, strong positive correlation. We then group each of these categorized correlation matrices according to the 10 non-redundant tetranucleotide combinations of Y/R bases, and for each entry selected the dominant mode to describe the subset (i.e. the most common situation shared by the individual tetranucleotides within a family).

### Statistics, graphics and molecular plots

The statistical analysis, including the Bayesian Information Criterion (BIC), Bayes Factor analysis, Helguerro's theorem, Kullback–Lieber divergence, PL and correlations measurements, as well as associated graphics, were obtained with R 3.0.1 statistical package (51), the MatLab R2016b package, numpy (52) and matplotlib (53). The molecular plots were generated using VMD 1.9 (54).

## RESULTS AND DISCUSSION

### Sources of uncertainty: the sequence library and the type of salt

**Figure 1.**
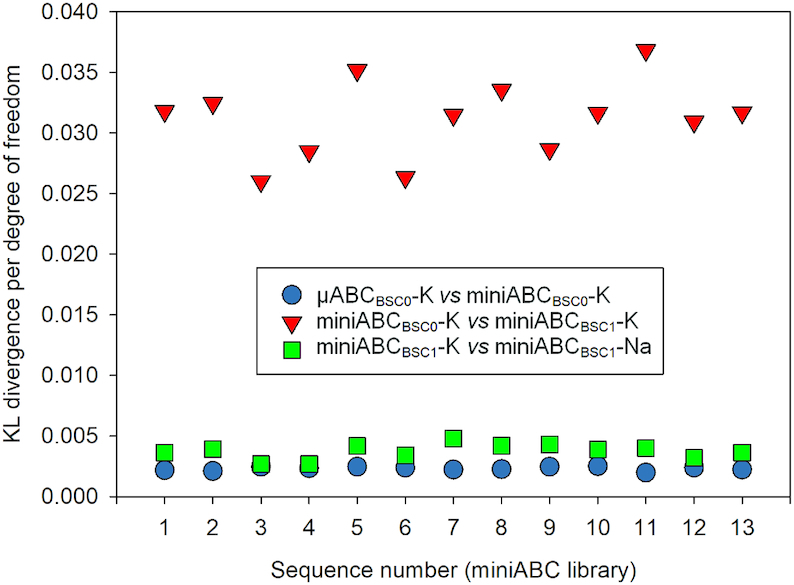
Symmetric Kullback–Leibler divergence per degree of freedom between Gaussian distributions, which is a combined measure of differences in values of first and second moments, for each of the thirteen oligomers in the miniABC training library, but for cgDNA model parameter sets fitted to different MD simulation protocols (see Materials and Methods and Supplementary Methods).

Before going into detail with a conformational analysis, we first considered the robustness of our results to changes in the choice of sequence library, because large differences would challenge the general validity of our conclusions. Fortunately, only one of the 1,632 distributions analysed (namely of 6 intra- plus 6 inter-basepair helical parameters for each of the 136 distinct tetranucleotides), showed significant differences (according to BIC-Helguerro analysis) depending on the choice of library (the previous μABC library, or the current miniABC library; see Supplementary Figure S1). Furthermore, no differences were found depending on the salt (see Supplementary Table S2 and raw data in https://mmb.irbbarcelona.org/miniABC/), which suggests that our results are robust to the choice between K and Na for the counter-ion. To gain additional confidence in the robustness of our results, we used the explicit form of Kullback–Leibler divergence available for Gaussian (i.e. multivariate normal) distributions to quantify three pairwise differences in cgDNA model predictions (see Materials and Methods, and Supplementary Methods) of the mean and covariance for each of the 13 miniABC library sequences for the four different parameter sets extracted from the μABC_BSC0_-K, miniABC_BSC0_-K, miniABC_BSC1_-K and miniABC_BSC1_-Na simulations. As can be seen in Figure 1, no significant difference arises from the change in the sequence library, nor from the difference between K and Na counter ions. However, the results are quite sensitive to the change in force field from PARMBSC0 to PARMBSC1. This is to be expected since the latest PARMBSC1 force field leads to a considerably more realistic representation of twist/roll and BI/BII distributions (see the analysis and discussion published elsewhere (9,25)), and to straighter average configurations of duplexes than those obtained from prior force fields. This can be confirmed by considering the differences between static and dynamic persistence lengths (as introduced elsewhere (55)) over a large ensemble of sequences (see Supplementary Figure S2).

### Strong anharmonic distortions do arise

One of the most important extreme deformations of DNA is the disruption of base pairing, which can be analyzed in detail by aggregating data over all instances of G:C and A:T basepairs. This allowed us to accumulate ensembles of independent 3D conformations, which added together reach the millisecond time scale. Terminal basepairs (G:C pairs in all the cases) showed open states (water molecules in between H-bonding Watson–Crick groups) in 1–2% of the total simulation time, with short average open lifetimes (around 3 ns, see Supplementary Table S3) in agreement with time-resolved Stokes shifts spectroscopy (56), but most probably too short to lead to isotope exchange signals in NMR experiments (57). The opening of central basepairs is less likely to occur (between 0.01% in G:C and 0.05% in A:T of the simulation time), but when it happens, the open state can survive considerably longer (up to 50 ns). Whether or not this time is sufficient to allow proton interchange with the solvent is unclear. Another example of a strong anharmonic deformation arising in our simulations is the temporary formation of a sharp kink (Supplementary Figure S3) associated with anomalous rise and roll (58,59) at an AA basepair step within a TAAA tetranucleotide belonging to a relatively long continuous stretch of A:T basepairs (seq. 9, see Supplementary Table S1). Very interestingly, this deformation (not previously seen in simulations) has been characterized before as one of the origins of bubbling and kinking in natural DNA (60,61), and correlates with the extreme flexibility of NTAN (where N = A, C, G, T) sequences described by NMR experiments (62) and MD simulations (63).

### Equilibrium distributions of basepair deformations are close to harmonic

**Figure 2.**
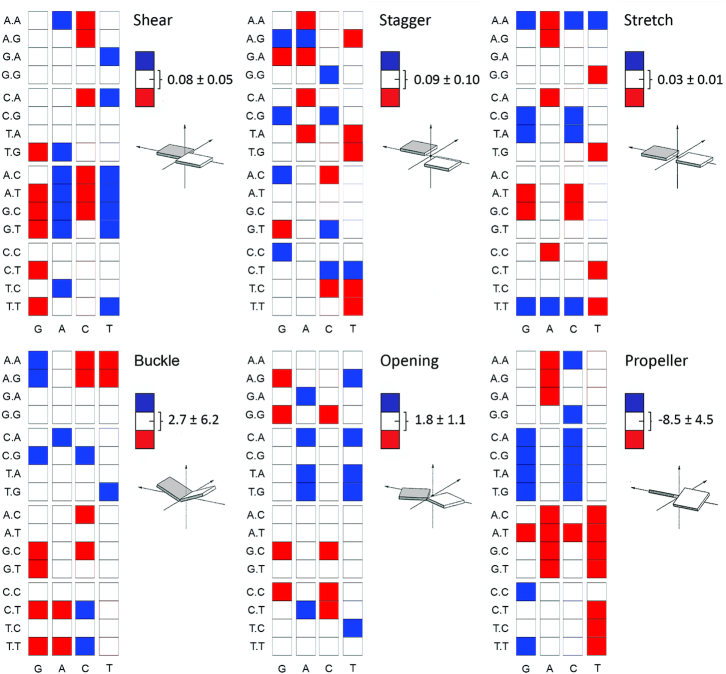
Average values of intra-basepair helical coordinates of the central base-pair (x-axis) in all possible 64 trinucleotide sequence contexts (y-axis). Results obtained from the miniABC_BSC1_-K simulations. The global averages (white) are over all sequence contexts and standard deviations reflect the variation among trinucleotide contexts. The blue squares mean that a specific base-pair has an average value above the global average plus one standard deviation, while red squares mean an average value below the global average minus one standard deviation.

A BIC analysis was carried out for the distributions of all six of the intra-basepair helical parameters at the central base pair in all 64 possible trinucleotide contexts. These distributions were all observed to be rather close to Gaussian, cf. Supplementary Figure S4, with the save for exceptionally rare events, as discussed in the last paragraph. Certainly no multi-peaked distribution was ever observed. Nevertheless the average value, or first moment, of each of the six intra-basepair parameters is strongly sequence-dependent to at least the trinucleotide sequence context, see Figure 2. Some qualitative rules on the sequence-dependent variation in the means can be observed. Shear values in G:C base pairs, when G is followed by Y are below average, while the opposite happens for A:T base pairs. Buckle in G:C shows large variations depending on the nature of the 3′-base of G, with an R leading to large positive buckles, and a Y leading to large negative buckles. Propeller also shows clear sequence effects, with A:T base pairs having a sizeable negative value when there is an R 5′ to the A, while propeller is close to zero for G:C basepairs within YGR trinucleotides.

### Equilibrium distributions of basepair step deformations are frequently strongly anharmonic

**Figure 3.**
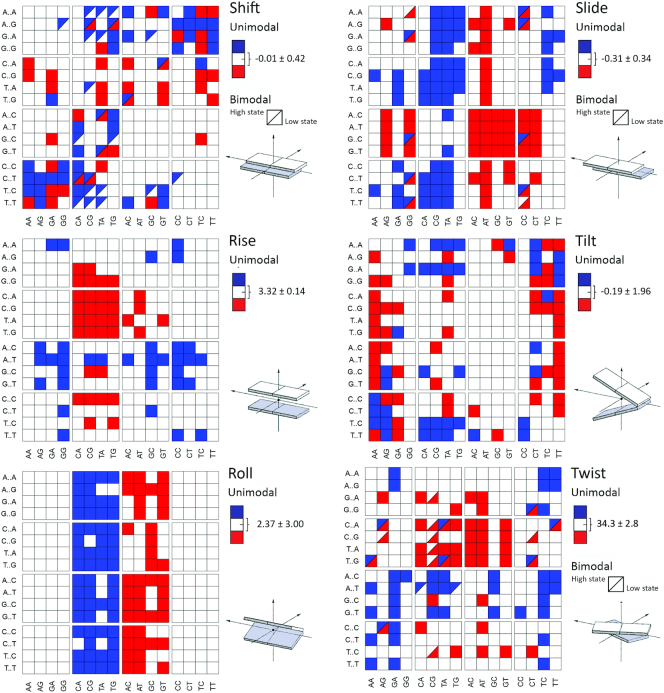
Average values of inter-basepair, or step, helical coordinates for the central junction (x-axis) in all possible 256 tetranucleotide contexts (y-axis). Results obtained from the miniABC_BSC1_-K simulations. Tetranucleotides classified as bimodal (half-square) are polymorphic (i.e. they sample two clear conformational substates). The global averages (white), exhibited on the legend at the right of each squared-plot, were computed from the weighted-averages obtained through BIC (see Materials and Methods and Supplementary Methods), and while the standard deviations reflect the variation among the tetranucleotide sequences that share the same central basepair step. The blue squares mean that a specific step has an average value above the global average plus one standard deviation, while red squares mean an average value below the global average minus one standard deviation.

Bi-normality (i.e. deviation from gaussianity) in the equilibrium distributions of the inter-basepair helical coordinates is common, but clear bimodality (i.e, the appearance of distinct multiple peaks) is observed in only 3% (miniABC_BSC1_-K^+^) to 5% (miniABC_BSC1_-Na^+^) of the inter-basepair helical parameter distributions (Figure 3 and Supplementary Figure S5). Bimodality appears systematically only for slide (several tetranucleotides containing a central GG step), shift (typically in a few tetranucleotides containing a YR central step) and twist (mainly in tetranucleotides containing central CG or AG steps). These conclusions are completely compatible with our prior analysis of PARMBSC0 simulations (see the μABC work (10), particularly Figure 8). There are few cases where bimodality affects simultaneously two or more helical parameters, for example, AGGA and GGGA are bimodal in shift and slide (in agreement with experimental data (64)) and ACGG, GCGA and GCGG are bimodal in shift and twist in agreement with results derived from the data mining of PDB structures (5). The central step of the GTAA tetranucleotide is the only case displaying bimodality in three helical parameters (shift, slide and twist) simultaneously. In general, shift bimodality is coupled with the appearance of high-shift values (>1 Å). The reverse situation was found for slide, where bimodality displaces the distribution to lower values. Finally, twist bimodality displays more complex behavior, as in some cases the second peak of the distribution occurs at lower than canonical values (<30°), while in others it is at high twist values (>40°). See Figure 3 and Supplementary Figures S6–S8 for a detailed analysis.

While inter-basepair, or junction, helical coordinates are frequently far from having a normal distribution, the first and second moments of their equilibrium distributions are still well defined, and can be approximated by evaluating the appropriate averages along our MD simulation time series, and over all instances of dinucleotide (or NN, nearest neighbor) or tetranucleotide (NNN, next nearest neighbor) contexts. Only a few general NN rules can be observed for the first moments: (i) As suggested by Calladine roll (YR) > roll (RY) and twist (RY) > twist (YR); (ii) YR basepair steps typically have higher than normal slide and roll; (iii) RY steps typically have lower than normal slide and roll and (iv) YY and RR steps have lower than normal tilt values. Any further rules concerning the average values of helical inter-basepair coordinates need to be formulated as the averages for the central junction or step in a specific tetranucleotide sequence context due to strong nonlocal sequence dependence, at least in part due to tetranucleotide dependent anharmonic effects (Figure 3 and discussion below).

### Backbone polymorphism

**Figure 4.**
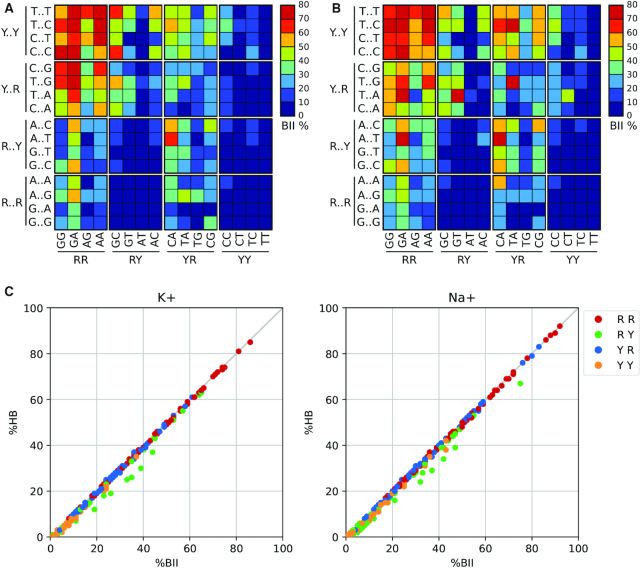
Sequence dependence of BII backbone conformations comparing K^+^ and Na^+^. (**A**) miniABC_BSC1_-K BII percentages. (**B**) miniABC_BSC1_-Na BII percentages. (**C**) Correlation between the percentage of BII (%BII, horizontal axis) and the occurrence of formation of the C–H···O H-bonds (%HB, vertical axis) at the central base step of each of the 256 possible tetranucleotide sequences, colour-coded according to the base type of the central base step.

The flexibility of DNA backbone is linked to rotations around seven torsion angles (α, β ,γ, δ, ϵ, ζ and χ, with δ in the present analysis being replaced by the sugar phase angle P), which in some cases move in a concerted way (e.g. α/γ and ϵ/ζ) between well-defined conformational sub-states. The best-studied of the coupled transitions is the so-called BI/BII transition, which occurs due to the concerted rotation of the ϵ/ζ torsions. BI→BII transitions are believed to be functionally relevant. They occur in some high-resolution crystal structures (65,66) and are also detected in ^31^P NMR spectra (67,68). Results in Supplementary Figure S9 show that the BII state is much more frequent than expected from simulations performed using previous force fields, matching NMR estimates for equivalent sequences (69). Very interestingly (see Figure 4, and Supplementary Tables S4 and S5), the BI/BII equilibrium is strongly dependent on the surrounding base sequence. For example, RR backbones (i.e. the ϵ and ζ torsions involving the phosphate between two purines) exhibit quite high BII percentages, especially in the presence of Y at the 5′ end of the corresponding tetranucleotide, while the YY backbones are typically biased towards the BI state, generating a strong asymmetry at RR:YY steps. While the general trends of BI/BII equilibria are robust with respect to changes in salt, a detailed analysis indicates the existence of subtle differences (5), which are especially visible for RR and YR basepair steps: in general, Na^+^ increases the total percentage of the BII state (Figure 4), but reduces its sequence-dependence, in perfect agreement with experimental data (70). As previously reported (4,5), we found a very strong correlation between BI→BII transitions and the formation of unconventional hydrogen bonds of the type CH—O, which are instrumental in mechanically coupling the movements detected in the backbone with those seen in the bases (see Figure 4, Supplementary Table S6).

In contrast to BI/BII dynamics, the α/γ conformational landscape is dominated by the canonical conformation, which, on average, represents ∼90% of the collected ensembles. Non-canonical conformers are more likely to appear in Na^+^ simulations than with K^+^ (Supplementary Tables S7 and S8). Transitions to non-canonical α/γ conformations are frequent, but the alternative states tend to have a short lifetime (on average we measured ∼500 transitions per μs per nucleotide, with an average residence time ∼5 ps). These brief transitions have little impact on the global conformational ensemble (9). No clear sequence-related rules can be determined for α/γ transitions, but, as expected, C and G nucleotides show longer-lived and more frequent α/γ transitions than A or T (8,9,71). Pseudo rotational Phase (P) angle analysis (Supplementary Figure S10) shows South (C2′-endo, ∼150°) conformations are dominant as expected, but East conformers are common, and sampling North states is not rare, especially for pyrimidines (9). As also expected, glycosidic torsions (χ) are always in the *anti* region (−180 to −90°), with purines sampling more frequently than pyrimidines the *high-anti* conformations (−90 to −30°; see Supplementary Figure S11). Finally, all nucleotides exhibit the same wide distribution for the β angle, spanning from 120° to 240°, with a strongly marked peak at the canonical value (180°) and a marginal population at ∼70° (g+, see Supplementary Figure S12), in good agreement with results from the data mining of X-ray structures (72).

### The choreography of correlated motions in the DNA

The movements of the DNA duplex often involve concerted changes in conformational degrees of freedom, generating a complex choreography. As an example, puckering (measured by the pseudo rotational angle P) and glycosidic torsions (measured by the χ angle) are tightly coupled, and the population of East and North puckering leads to a marked displacement of χ to lower values (Supplementary Figure S13). Furthermore, χ and P torsions are coupled to the ϵ/ζ changes in a sequence-dependent manner (Supplementary Figure S14). Thus, in purines, the population of the BII state is coupled to a displacement of puckering to the East (P) and (χ) *high-anti* regions, while in pyrimidines the population of BII conformers leads only to a slight displacement to the *high-anti* region, without significant puckering changes.

When the conformational analysis is carried out at the basepair level, a pattern of sequence-dependent correlated movements emerges. All distinct trinucleotides show moderate-to-high correlations in shear-opening, shear-stretch, and stagger-buckle. The pattern of correlation is less clear for the remaining intra-basepair parameters, although several trinucleotides show stretch-opening correlations (Supplementary Figure S15). A more complex sequence-dependent picture of correlated movements can be obtained by analyzing the inter-basepair helical parameters (Supplementary Figure S16). For example, mild to strong correlations are found in shift-tilt, slide-twist, rise-tilt, shift-slide, and shift-twist movements for RR steps. For RY, weaker correlations can be found (depending on the tetranucleotide sequence-environment) in shift-tilt, slide-rise and roll-twist. Finally, YR basepair steps may exhibit moderate to strong correlations for shift-tilt, slide-twist, rise-twist and roll-twist (Supplementary Figure S16). Interestingly, for all the tetranucleotides, shift-slide and roll-twist always show negative correlations, while shift-tilt and slide-twist always show positive correlations. As expected, correlations also emerge when combining inter- and intra-helical parameters in the same analysis. Thus, a significant number of tetranucleotides show moderate to strong correlations of opening with shift, buckle with rise, and stagger with tilt (data not shown). It is also worth noting that the network of correlations extends to neighboring steps. As an example, twist in the central YR step of XYRR tetranucleotides is highly correlated with slide in the adjacent RR step (5,10), which again stresses the limitations of simple nearest neighbors interpretations of DNA conformational mechanics, and points the way to coarse grain models such as cgDNA (48), that encompass longer range coupling, with associated longer-range sequence-dependence of the observed means and many non-vanishing covariances.

Lastly, backbone and basepair conformations are connected in a complex way, with ϵ/ζ (BI/BII) being the major determinant of the polymorphism. Very often, tetranucleotides showing a simultaneous sampling of BI and BII conformations are those with bimodality in some helical parameter at the same step (70% of the bimodal inter-basepair helical parameters occur in steps with bimodal BI/BII distributions, see Figure 3 and Supplementary Tables S4 and S5). The BI/BII state also correlates with inter-basepair helical coordinates in neighboring junctions, explaining part of the geometrical constraints postulated by Calladine. For example, the increase in the percentage of BII at the central junction of a given tetranucleotide correlates with larger shift values at the same junction for all sequences (Supplementary Figures S17) and is also coupled to lower twist and slide values. The BI/BII ratio at a junction *i* also correlates with shift, twist and slide values at step *i* + 1 and *i* – 1 (Supplementary Figures S18 and S19), highlighting the subtle mechanical coupling between backbone and basepair step conformations within DNA (72).

**Figure 5.**
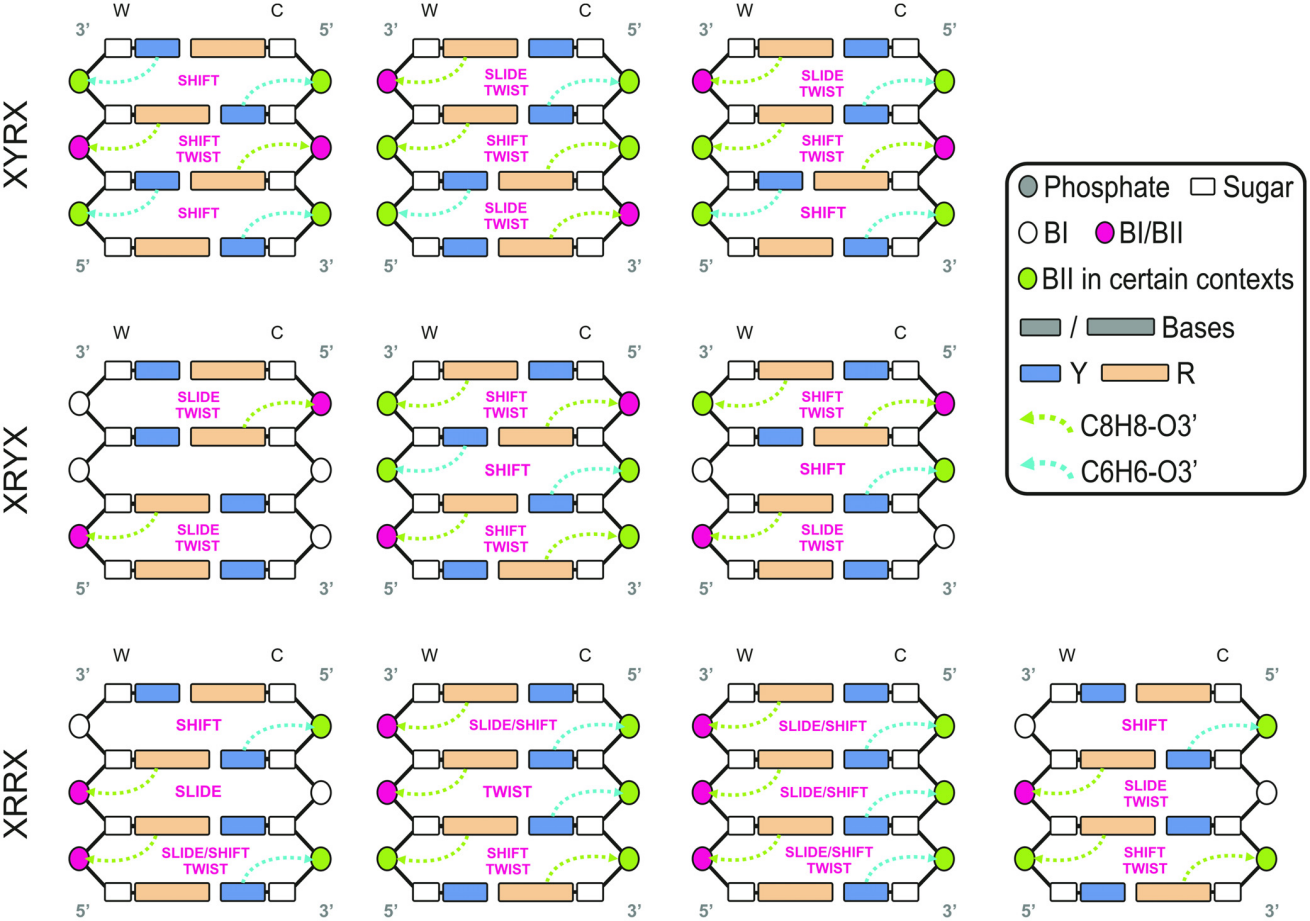
Schema of the polymorphic, or multi-well, landscape exhibited by B-DNA at the tetranucleotide level expressed in the purine (R)/pyrimidine (Y) alphabet, for which only 10 distinct combinations exist, but which still distinguish all possible behaviors. The only helical coordinates that exhibited multi-modality are shift, slide and twist, and each junction in the figure is marked with which coordinates can be multi-modal in it. There is a very high correlation between the occurrence of multi-modality and the formation of a noncanonical hydrogen bond in either the same or a neighboring junction, along with its associated BI/BII backbone transition (see text).

All the observations made above can be unified in a global flexibility scheme for B-DNA (Figure 5), showing that all basepair junctions contain potentially polymorphic elements (BI/BII, shift, slide, or twist) that can lead to bimodal behavior depending on the specific tetranucleotide environment. The analysis we have carried out leads to a scheme with strong predictive power at the tetranucleotide level. As a single example, we can now say with confidence that when the choice of X and Y within an XYRY tetranucleotide leads to bimodality, this will be expressed in shift and twist, coupled with a low-to-moderate percentage of BII in the Watson strand. In contrast, when XRRX tetranucleotides are considered, bimodality will show up in either shift, slide or twist (or a combination thereof), coupled with a moderate-to-high percentage of BII in the Watson strand of the central junction.

### Calladine–Dickerson rules and beyond

Calladine's principles were based on steric hindrance and stacking, which constrained a few helical parameters to some specific conformations (13). Dickerson transformed Callandine's principles in four simple sum functions (Σ_1_ for twist), by which the expected local variation in twist, roll, torsion angle δ, and propeller twist could be explained from the DNA sequence (14). The Calladinec–Dickerson rules reproduced DDD (Drew–Dickerson Dodecamer), although the predictive power was limited since the value corresponding to one unit of Σ, and the center of the sum functions (i.e. 2.1° and 35.6° for Σ_1/DDD_ respectively) could only be set *a posteriori* of knowing the 3D structure (14).

Modern force fields for DNA were fine-tuned over decades (20,24,30) and are now able to reproduce accurately helical conformations and backbone substates (25). Combined with a suitable library of sequences, the first moment (averages) of the equilibrium distributions of helical coordinates (36 distinct parameters if a basepair step is considered) for any sequence-dependent conformation could be predicted. This removes the need of previous knowledge of the 3D structure, extending Calladine–Dickerson's rules to any possible helical parameter and adding on top the polymorphic landscape of the backbone, the bases, and all the cross-correlations. We generate here ensembles for all the 256 possible tetranucleotide combinations from the 136 unique tetranucleotides found in the miniABC library, by inverting the shift and tilt values of the central step in complementary sequences (i.e. shift_AGTT_ = −shift_AACT_). Similarly, ensembles for the 64 possible trinucleotides are generated from the first occurrence of the 32 unique trinucleotides with central purine.

**Figure 6.**
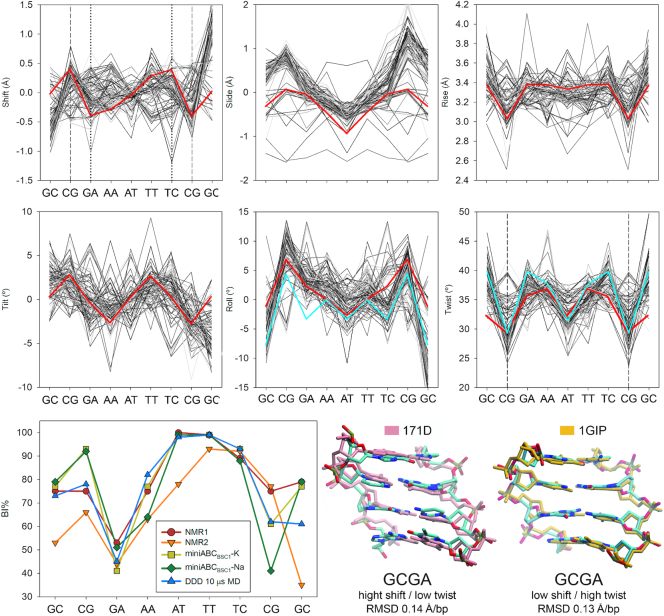
Comparison between experimental structures (X-ray and NMR) determined for the Drew−Dickerson Dodecamer (9), and conformations predicted by using the MD datasets produced herein. The six intra-basepair parameters were predicted (red lines) and compared with all the experimental structures (gray lines) and Calladine–Dickerson prediction for Roll and Twist (cyan line). Vertical dashed lines represent predicted binormal/bimodal steps, while vertical dotted lines represent steps with clear multi-peaked distributions although not bimodal according to Helguerro (see Materials and Methods). In the last row, predicted BI% (yellow and green) were compared with ^31^P-NMR gold-standard measurements and multi-microsecond long MD simulation of the same sequence using PARMBSC1 force field (9). NMR1 stands for the work by Schwieters *et al.* (74), and NMR2 from Tian *et al.* (68). 3D superposition showing the capability of the miniABC library and our set of rules to predict two conformational substates observed experimentally for DDD at the highly polymorphic GCGA tetranucleotide (NMR structures with PDB id: 171D, and 1GIP). The RMSD was measured after aligning the backbones between the experimental structures and the two substates captured by the miniABC library.

Experimental validation of our extended set of rules to predict B-DNA conformation based on the sequence is still difficult to achieve, as DDD is the only sequence that was determined experimentally enough times, using significantly different techniques, protocols, and laboratory conditions, to have a view, yet limited, of the structural heterogeneities of a given isolated B-DNA (9). Moreover, it's the only sequence for which two independent ^31^P-NMR experiments were performed, and from which accurate BI% were obtained (68,73). Thus, we retrieve 93 structures from the PDB (9), plus the two ^31^P-NMR experiments to compare the results of our predictive rules on the DDD sequence (Figure 6). Helical conformations predicted in terms of the inter-basepair parameters were in excellent agreement with the experimental ensemble and Calladine–Dickerson rules (see roll and twist in Figure 6), reproducing sequence-dependent features of this prototypical B-DNA sequence. Moreover, we correctly predicted base polymorphisms in shift and twist (5,10,18) and most importantly the backbone substates, in particular, BI/BII (Figure 6). Notwithstanding the foregoing, using our webserver (https://mmb.irbbarcelona.org/miniABC/), the average inter-basepair helical parameters of other B-DNA sequences could be predicted in few seconds giving results in good agreement with experiments determined by NMR and X-ray (Supplementary Figures S20-S27), and comparable to the ones obtained by microsecond long simulations (Supplementary Figures S26 and S27) (25).

The polymorphisms presented thorough this contribution, are dynamic structural heterogeneities that follow two main rules: (i) The crankshaft motion of the DNA backbone that precludes the existence of high BII% in two consecutive junctions (74), and explains the correlations found in consecutive helical parameters (Supplementary Figures S18 and S19) (63). (ii) The existence of conformational frustration, which implies that two or more bimodal elements cannot co-exist at the same time in a given substate. In this sense, when using the information from the second moment (covariance) a dynamic rule appears: If two consecutive high BII (>40%) backbones are predicted in the same strand, the one with the highest value should be kept in BII while the other remains in the BI substate (according to the corresponding value of each peak found by BIC in bimodal distributions). These simple dynamic rules allow to correctly model the structural heterogeneities of B-DNA, capturing the subtle conformational differences found by solution NMR structures for the DDD sequence (Figure 6).

## CONCLUSIONS

The analysis of numerous molecular dynamics trajectories obtained with an accurate, last generation, force field has allowed us to derive some general rules concerning the equilibrium conformation distribution of B-DNA, which represent a significant step beyond Calladine–Dickerson earlier propositions. Specifically, we are now able to predict when significantly anharmonic distributions will arise as a function of the tetranucleotide sequence context:

- The first and second moments (average and covariance) of the equilibrium distributions of helical coordinates for DNA can only be understood in terms of nonlocal sequence-dependence contexts, to at least the trinucleotide level for intra-basepair coordinates, and the tetranucleotide level for inter-basepair coordinates.
- A harmonic model of DNA dynamics will not be able to accurately predict third and higher moments of the equilibrium distribution because significant anharmonic movements arise frequently. In fact, the distribution of many inter-basepair coordinates is significantly binormal and, in a non-negligible number of cases, actually bimodal (i.e. multi-peaked). Such bimodality and the relative population of corresponding local minima of the free energy is dependent on the tetranucleotide context. Slide for GG, twist for CG and AG, and shift for YR are the most common steps and helical coordinates exhibiting bimodality, with the tetranucleotides most commonly enhancing bimodality being AGGA, GGGA, ACGG, GCGA, GCGG and GTAA.
- Backbone torsional changes are coordinated in pairs (α/γ, P/χ and ϵ/ζ). Movements in α/γ lead to the generation of short-lived non-canonical states, which can, however, be populated in the presence of ligands, as it was previously observed for protein-DNA complexes (7). Changes in sugar puckering to the East region leads to lower χ values, while coordinated changes in the ϵ/ζ pair lead to the BI/BII polymorphism with coupled impacts on helical parameters. Both ϵ/ζ and P/χ couplings exhibit sequence dependence.
- The BI/BII conformational change is coupled to the cationic atmosphere surrounding DNA, and to the formation of non-canonical CH—O base-backbone hydrogen bonds. BI/BII transitions are especially prevalent for YRRX sequences and often are associated with bimodality in helical coordinate distributions at the basepair step level. They are a major source of polymorphism in B-DNA. In general, the population of the BII state is coupled to large shift, and low slide and twist at the same junction, but distant and more complex correlations exist between BI/BII conformational states and the helical conformation of neighbouring steps.
- Helical parameters at a given step are not independent but show a complex backbone-mediated pattern of dependencies. For example, shift-slide and roll-twist always show negative correlations, while shift-tilt and slide-twist always show positive correlations. On the contrary, correlations between slide-rise and tilt-roll vary as a function of base sequence. Moreover, helical coordinate correlations may extend to neighboring base pairs as a function of the local sequence.
- Although the differences are subtle, the polymorphic landscape delivered by the simulations in NaCl is slightly richer: (i) non-canonical α/γ conformers are more likely to appear in Na^+^ simulations than with K^+^; (ii) in general, Na^+^ increases the total percentage of the BII substate, but reduces its sequence-dependence; (iii) the appearance of clear bimodality in the inter-basepair helical coordinates is more common in Na^+^ simulations than with K^+^ (3% in miniABC_BSC1_-K^+^ versus 5% in miniABC_BSC1_-Na^+^).
- Calladine's principles and Dickerson's sum functions for twist/roll/δ/propeller can now be transformed into quantitative predictions for all the structural features (helical conformations and backbone substates) of canonical DNA sequences. These extended rules have been implemented in a web server that predicts the average conformation of any B-DNA sequence, in terms of the average helical parameters, base and backbone polymorphisms, and P/χ conformations (see https://mmb.irbbarcelona.org/miniABC/).
- Furthermore, using the predictive cgDNA coarse-grained model (and its dinucleotide dependent parameter sets fit to MD simulations), the nonlocal sequence-dependent first (average) and second (covariance) helical coordinate moments can be computed interactively for an arbitrary sequence on the cgDNAweb (75) server http://cgdnaweb.epfl.ch/, including interactive visualization of the expected or ground state conformation.

## Supplementary Material

gkz905_Supplemental_File
