# Supplementary material for: The static and dynamic structural heterogeneities of B-DNA: extending Calladine–Dickerson rules
Source: Nucleic Acids Res. 2019 Oct 18;47(21):11090–102. doi: 10.1093/nar/gkz905 (PMC6868377; doi:10.1093/nar/gkz905)
Supplement: gkz905_Supplemental_File [file gkz905_supplemental_file.pdf]

SUPPLEMENTARY MATERIAL

THE STATIC AND DYNAMIC STRUCTURAL HETEROGENEITIES OF B-DNA: EXTENDING CALLADINE-DICKERSON RULES

Pablo D. Dans[a,b,1], Alexandra Balaceanu[a,2], Marco Pasi[c,d,2], Alessandro S. Patelli[e,2], Daiva Petkevičiūtė[e,f,2], Jürgen Walther[a,2], Adam Hospital[a], Genís Bayarri[a], Richard Lavery[d], John H. Maddocks[e,1], and Modesto Orozco[a,g,1]

[a]Institute for Research in Biomedicine (IRB Barcelona). The Barcelona Institute of Science and Technology. Baldiri Reixac 10-12, 08028 Barcelona, Spain.
[b]Department of Biological Sciences, University of the Republic (UdelaR), CENUR Gral. Rivera 1350, 50000 Salto, Uruguay.
[c]LBPA, École normale supérieure Paris-Saclay, 61 Av. du Pdt Wilson, Cachan 94235, France.
[d]Bases Moléculaires et Structurales des Systèmes Infectieux, Univ. Lyon I/CNRS UMR 5086, IBCP, 7 Passage du Vercors, Lyon 69367, France.
[e]Institute of Mathematics, Swiss Federal Institute of Technology (EPFL), CH-1015 Lausanne, Switzerland.
[f]Faculty of Mathematics and Natural Sciences, Kaunas University of Technology, Studentų g. 50, 51368 Kaunas, Lithuania.
[g]Department of Biochemistry and Molecular Biology. University of Barcelona, 08028 Barcelona, Spain.

[1]To whom correspondence should be addressed:
Prof. Pablo D. Dans, Tel: +34 934039073, Email: pablo.dans@irbbarcelona.org / pablo.dans@unorte.edu.uy; or
Prof. John H. Maddocks, Tel: +41 216932762, Email: john.maddocks@epfl.ch; or
Prof. Modesto Orozco, Tel: +34 934037155, Email: modesto.orozco@irbbarcelona.org.

[2]These co-authors equally contributed to this work and were alphabetically sorted.

SUPPLEMENTARY METHODS

Bayesian Information Criterion (BIC), Bayes Factors, and the Helguerro's theorem. We used the BIC methodology to determine the optimal number of Gaussian functions needed to fit a given distribution. This is done by finding the set of parameters that minimizes the BIC values (the model with the lower BIC is chosen) according to(1):

$$-2lnp(x|k) \approx BIC = -2\ln(L) + kln(n)$$

Where *x* are the observed data, *k* is the number of free parameters to be estimated, and *p(x|k)* is the probability of the observed data given the number of parameters, or, in other words, the likelihood of the parameters given the dataset. *L* is the maximized value of the likelihood function for the estimated model, and *n* is the number of data points in *x* (the number of observations). In this work we limit the BIC to considering a maximum of two Gaussians, leading to the classification of each distribution as uninormal (fitted with one Gaussian) or binormal (fitted with a combination of two Gaussians).

The Bayes Factors that can be extracted from the BIC analysis were used to determine the strength of the evidence in favour of the model chosen by BIC(2, 3). This leaded to a third classification labelled as "insufficient evidence", when either of the two models determined with BIC (uninormal or binormal) couldn't be statistically supported.

Finally, when there was sufficient evidence to favour a binormal fitting, we used an extension of the Helguerro's theorem(4, 5) to define the modality of the distribution and distinguish the cases where the two peaks of the fitted Gaussians are close together from those where they are significantly separated. This is the most important distinction in terms of understanding DNA dynamics. In the first case, for practical purposes, the use of a single Gaussian distribution may often be justified to represent the data (the overall distribution may be interpreted as binormal-unimodal), while it cannot be used to estimate higher moments in the second multi-peaked case (binormal-bimodal distributions). For a given parameter, we defined an inter-basepair, or intra-basepair as polymorphic from the structural point of view, when a given distribution was classified using these three approaches as binormal-bimodal.

Kullback-Leibler (KL) divergence between configuration distributions. For each MD simulation we fit a Gaussian or multi-variate normal distribution on the helical coordinates by estimating a mean shape vector $\hat{w}$ and a stiffness, or inverse covariance matrix K, from the MD time series. (This Gaussian is in dimension 12N-6 for a fragment with N base pairs, so dimension 210 for the case N=18 considered here.) The KL

divergence(6) is a convenient way to quantify the difference between two probability distributions. When both distributions are Gaussian with mean vectors $\hat{w}_1$, $\hat{w}_2$ and inverse covariance matrices $K_1$and K_2, then the divergence can be explicitly evaluated as:

$$D_{12} = \frac{1}{2}\left[K_1^{-1}: K_2 - \ln\left(\frac{\det K_2}{\det K_1}\right) - I: I\right] + \frac{1}{2}(\hat{w}_1 - \hat{w}_2) \cdot K_2(\hat{w}_1 - \hat{w}_2),$$

Where a colon denotes the standard Euclidean inner product for square matrices and I denotes the identity matrix of the same dimension as K_1 and K_2. The second term of this expression is interesting to look at separately: it quantifies the difference in expected shapes, weighted by one of the inverse covariance, and is equal to the square of the Mahalanobis distance:

$$M_{12} = \frac{1}{2}(\hat{w}_1 - \hat{w}_2) \cdot K_2(\hat{w}_1 - \hat{w}_2),$$

Both KL divergence and Mahalanobis distance are non-symmetric, but here we chose to report the symmetrized values: $D = \frac{1}{2}(D_{12} + D_{21})$ and $M = \frac{1}{2}(M_{12} + M_{21})$. To give a meaning to values of the KL divergence, the KL values were scaled by 12N-6 (being N the number of base-pairs in each oligomer), obtaining in this way a divergence per degree of freedom.

<u>cgDNA calculation of DNA Persistence Length</u>. The cgDNAmc code(7) allows efficient generation of ensembles of configurations over ensembles of sequences, so that the possible range of values of various expectations can be examined as the sequence of the DNA duplex varies. One standard set of expectations to compute is tangent-tangent correlations along the duplex in order to determine the associated decay rate or persistence length ℓ_p along a given fragment. The persistence length ℓ_p is often taken as an overall proxy for the stiffness of the duplex, with longer persistence length indicating greater stiffness. However it is known (see *e.g.* the discussion in ref (7)) that the value of ℓ_p depends on both the stiffness of the duplex and on its intrinsic curvature, with bent sequences having lower persistence lengths. For this reason ℓ_p is sometimes called apparent persistence length. A sequence-dependent dynamic persistence length ℓ_d was introduced(7), which largely eliminates dependence on intrinsic curvature. Thus ℓ_d is a better proxy for an overall stiffness, while the difference (ℓ_d - ℓ_p) is an overall measure of how intrinsically bent the duplex is. Fig S2A provides spectra (or histograms) of possible values of both ℓ_p and ℓ_d for 10K sequences according to a cgDNA model parameter set fit to MD simulations of the miniABC library using the PARMBSC0 MD potentials. The range of variation in ℓ_d is small compared to that of ℓ_p, and it can be verified that all exceptionally low values of ℓ_p correspond to highly bent sequences. The

same data for the same 10K sequences, but for a cgDNA model parameter set fit to MD simulations of the miniABC library using the PARMBSC1 MD potentials is shown in Fig S2B. The fact that the spectra of dynamic persistence lengths ℓ_d shifts to the right indicates that the PARMBSC1 potentials lead to duplexes that are slightly stiffer than for PARMBSC0, while the fact that the spectra of apparent persistence lengths has a smaller tail on the left indicates that PARMBSC1 leads to duplexes that have smaller intrinsic bends than for PARMBSC0. Figure S2 also provide the values of apparent and dynamic persistence lengths for the six independent dinucleotide tandem repeats poly(XZ). As such sequences are very straight, their apparent and dynamic persistence lengths are all very close. And for both the PARMBSC0 and PARMBSC1 parameter sets the sequence poly(AA) is the high outlier among all sequences, with poly(AT) being by far the low outlier for ℓ_d among all sequences.

SUPPLEMENTARY TABLES

Table S1. DNA sequences in the miniABC library.

Seq. number	Watson strand (5'-3' direction)
1	GCAACGTGCTATGGAAGC
2	GCAATAAGTACCAGGAGC
3	GCAGAAACAGCTCTGCGC
4	GCAGGCGCAAGACTGAGC
5	GCATTGGGGACACTACGC
6	GCGAACTCAAAGGTTGGC
7	GCGACCGAATGTAATTGC
8	GCGGAGGGCCGGGTGGGC
9	GCGTTAGATTAAAATTGC
10	GCTACGCGGATCGAGAGC
11	GCTGATATACGATGCAGC
12	GCTGGCATGAAGCGACGC
13	GCTTGTGACGGCTAGGGC

Table S2. Sequence-averaged conformational parameters obtained from the different miniABC simulations.[a]

	miniABC$_{BSC0}$-K		miniABC$_{BSC1}$-K		miniABC$_{BSC1}$-Na	
Parameter	Average	SD	Average	SD	Average	SD
Shear (Å)	0.02	0.30	0.02	0.30	0.02	0.30
Stretch (Å)	0.03	0.12	0.03	0.12	0.03	0.11
Stagger (Å)	0.06	0.40	0.10	0.38	0.10	0.38
Buckle (°)	0.8	10.8	1.5	9.9	1.6	9.7
Propeller (°)	-12.0	8.2	-9.0	8.1	-9.3	8.2
Opening (°)	2.2	4.5	1.8	4.3	1.8	4.2
Xdisp (Å)	-1.77	1.52	-0.88	1.36	-0.64	1.43
Ydisp (Å)	0.03	1.27	0.00	1.13	-0.01	1.17
Inclination (°)	8.2	7.1	4.0	6.6	2.8	7.0
Tip (°)	0.2	6.7	0.3	6.3	0.3	6.4
Shift (Å)	-0.03	0.69	-0.03	0.80	-0.04	0.83
Slide (Å)	-0.51	0.62	-0.29	0.55	-0.22	0.55
Rise (Å)	3.32	0.32	3.32	0.30	3.32	0.29
Tilt (°)	-0.3	4.3	-0.3	4.4	-0.3	4.5
Roll (°)	4.5	5.8	2.4	5.7	1.7	5.8
Twist (°)	32.1	5.6	34.4	5.5	34.7	5.3
α (°)	-71.1	13.9	-72.1	15.4	-72.3	15.4
β (°)	170.3	13.8	167.8	21.0	166.9	21.2
γ (°)	56.3	12.3	55.0	18.9	55.0	19.1
δ (°)	119.4	21.3	135.3	15.5	136.2	14.7
ε (°)	-167.4	25.4	-160.4	25.8	-158.6	27.1
ζ (°)	-94.1	33.5	-111.4	41.6	-113.8	43.8
χ (°)	-120.5	20.2	-112.1	17.0	-111.2	16.9
Phase (°)	128.3	37.6	151.4	26.5	152.3	25.0
Amplitude (°)	38.4	7.0	41.6	6.6	41.8	6.6

[a] Capping base pairs were removed from the analysis. For the dihedral angles only the Watson strand was considered.

Table S3. DNA breathing and fraying. Base opening statistics based on the analysis of the WC hydrogen bonds.

	Loss of one Hbond [a]		Loss of two Hbonds		Loss of three Hbonds		Solvent exchange[b]	
	Occ.[c] (%)	<t½>[d] (ns)	Occ. (%)	<t½> (ns)	Occ. (%)	<t½> (ns)	Occ. (%)	<t½> (ns)
				K+Cl-				
C:G bp terminal	3.73	0.099	2.55	0.754	1.73	1.332	2.14	3.436
C:G bp terminal(-1)[e]	0.33	0.327	0.01	15.53	<0.01	---	<0.01	---
C:G bp central	0.45	0.251	0.03	10.47	0.01	315.2	0.01	149.5
A:T bp central	1.67	0.089	0.06	7.700	---	---	0.03	41.54
				Na+Cl-				
C:G bp terminal	2.81	0.095	1.57	0.761	0.87	2.209	1.20	3.552
C:G bp terminal(-1)	0.38	0.288	0.01	14.39	<0.01	---	<0.01	.---
C:G bp central	0.52	0.222	0.03	8.651	<0.01	---	<0.01	---
A:T bp central	1.59	0.094	0.04	8.963	---	---	0.01	62.49

[a] We consider a hydrogen bond broken when the distance between the heavy atoms involved in the Watson-Crick interactions was greater than 3.5 Å. [b] Solvent exchange refers to base openings where at least one donor-acceptor distance of WC hbonds is larger than 6 Å. These large separations allow water molecules to interact directly with the base, and eventually exchange protons with imino groups of the bases. [c] Occ. stands for occurrence in %. [d] Average open base lifetime. [e] Refers to the C:G base-pair prior to last (residue numbers 2:35 and 17:20), see Table S1.

Table S4. BII percentages for all the 256 tetranucleotides obtained from miniABC$_{BSC1}$-K.

	GG	GA	AG	AA	GC	GT	AT	AC	CA	TA	TG	CG	CC	CT	TC	TT
T..T	52	74	64	74	65	44	11	57	49	49	19	47	24	9	14	1
T..C	66	86	40	81	45	39	6	40	58	41	22	39	37	11	15	2
C..T	56	62	56	70	45	6	2	13	42	30	24	45	23	2	10	3
C..C	72	86	37	53	64	23	5	40	36	41	22	24	9	5	24	1
C..G	62	71	36	64	23	19	4	13	24	26	27	18	8	2	11	1
T..G	65	75	47	50	53	33	11	24	30	49	15	26	14	8	7	2
T..A	45	66	31	43	35	35	6	13	32	14	14	28	15	6	7	1
C..A	40	59	25	50	49	26	5	11	18	9	12	20	14	5	5	0
A..C	19	51	24	29	16	5	1	15	53	30	13	46	13	1	11	1
A..T	12	46	8	23	15	5	1	17	61	28	9	24	8	1	10	1
G..T	13	38	13	23	8	2	0	5	31	36	12	15	3	1	9	1
G..C	34	56	11	19	13	4	1	3	39	28	14	21	9	1	6	1
A..A	23	46	8	23	9	2	1	6	36	12	18	22	10	1	8	0
A..G	33	59	21	26	9	2	1	5	30	41	30	27	8	1	7	1
G..A	14	37	8	13	6	2	0	4	10	9	4	7	4	0	3	0
G..G	22	38	15	18	6	4	1	1	27	11	7	31	6	1	3	1

Table S5. BII percentages for all the 256 tetranucleotides obtained from miniABC$_{BSC1}$-Na.

	GG	GA	AG	AA	GC	GT	AT	AC	CA	TA	TG	CG	CC	CT	TC	TT
T..T	67	78	67	92	55	33	7	41	58	53	22	51	36	18	15	2
T..C	72	88	52	90	46	42	9	42	67	80	34	59	43	14	17	3
C..T	62	64	59	69	29	7	1	10	52	43	28	51	34	7	14	3
C..C	65	86	54	54	49	21	6	30	49	57	27	41	14	8	25	2
C..G	45	51	34	59	21	37	5	7	38	18	19	19	20	5	16	2
T..G	54	65	34	63	47	47	8	17	29	76	21	22	21	11	12	3
T..A	50	70	15	45	34	75	12	3	37	17	21	27	20	43	7	2
C..A	50	49	35	47	44	32	6	9	20	5	14	36	25	10	7	1
A..C	31	54	39	39	10	4	1	19	55	40	14	52	19	0	12	1
A..T	23	72	14	30	14	4	1	21	83	23	5	24	10	1	6	1
G..T	25	36	23	36	6	3	0	5	41	30	15	30	5	1	7	1
G..C	44	57	26	23	14	4	1	4	50	31	12	32	9	1	4	1
A..A	26	49	18	26	11	4	1	7	50	13	16	29	11	1	8	0
A..G	32	46	21	28	8	2	1	6	22	29	25	21	9	1	8	0
G..A	21	34	17	16	5	4	1	5	20	2	4	8	5	0	2	0
G..G	29	30	21	15	6	7	1	2	27	9	8	27	8	2	4	1

Table S6. Pearson correlation coefficients between BII% and the formation of the C-H···O H-bond.

	BII% vs C8-H8···O3’		BII% vs C6-H6···O3’		
Set	RR	YR	RY	YY	Total
miniABC$_{BSC1}$-K	1.000	0.999	0.994	0.996	0.998
miniABC$_{BSC1}$-Na	1.000	0.999	0.995	0.997	0.998

Table S7. Percentages of α/γ torsions in the canonical sub-state (characterized by α in g- and γ in g+) for all the 256 tetranucleotides obtained from miniABC$_{BSC1}$-K.

Flanks \ Step	GG	GA	AG	AA	GC	GT	AT	AC	CA	TA	TG	CG	CC	CT	TC	TT
T..T	97	97	90	98	98	99	99	96	90	93	98	97	96	98	94	96
T..C	97	95	98	95	91	87	97	97	97	98	99	96	94	98	99	98
C..T	97	98	98	97	99	89	93	98	94	97	99	94	99	96	97	99
C..C	95	94	97	97	96	98	98	99	90	97	97	89	95	96	90	92
C..G	98	93	97	97	94	97	98	98	96	96	97	97	99	98	95	96
T..G	97	90	95	97	97	98	97	98	89	95	99	87	98	95	95	100
T..A	97	97	96	98	98	97	98	97	98	99	99	99	99	96	97	94
C..A	97	92	98	91	98	97	98	98	96	99	95	95	91	99	99	96
A..C	97	86	98	96	99	97	97	97	97	99	92	90	89	96	99	97
A..T	96	92	95	94	96	95	98	99	98	99	99	99	97	89	97	94
G..T	97	93	97	95	99	99	90	89	97	99	83	94	99	96	94	97
G..C	92	96	93	91	97	83	99	83	95	97	97	91	89	99	98	98
A..A	97	97	64	93	96	97	98	98	95	100	99	97	99	98	98	100
A..G	95	97	97	96	97	98	98	98	98	98	96	96	97	92	99	100
G..A	91	96	94	98	100	99	99	91	99	99	100	99	96	99	87	96
G..G	69	92	89	98	99	93	96	99	91	90	90	75	99	94	95	95

Table S8. Percentages of α/γ torsions in the canonical sub-state (characterized by α in g- and γ in g+) for all the 256 tetranucleotides obtained from miniABC$_{BSC1}$-Na.

Flanks \ Step	GG	GA	AG	AA	GC	GT	AT	AC	CA	TA	TG	CG	CC	CT	TC	TT
T..T	97	97	98	99	97	95	95	71	92	84	98	96	99	99	99	98
T..C	86	97	90	98	97	70	97	98	95	96	99	95	99	87	93	99
C..T	96	97	96	94	98	96	97	98	98	92	87	79	98	99	99	96
C..C	96	91	95	86	95	98	85	99	95	93	82	97	94	96	99	95
C..G	94	95	95	97	95	97	95	99	95	99	92	99	99	99	98	98
T..G	94	93	88	96	87	93	79	97	92	99	92	90	97	92	90	99
T..A	95	97	98	96	95	98	98	92	95	97	97	96	97	91	87	100
C..A	94	95	97	96	96	99	87	98	97	96	91	93	95	92	99	99
A..C	82	97	97	97	97	96	98	97	99	97	89	99	99	100	96	100
A..T	98	99	82	98	97	98	98	99	95	99	98	100	97	97	95	98
G..T	87	95	97	98	93	96	98	98	95	92	97	92	96	98	97	95
G..C	97	94	92	98	96	98	97	96	97	97	93	98	97	99	94	93
A..A	93	97	98	98	98	88	97	98	95	96	99	99	99	67	99	96
A..G	94	94	98	98	92	98	94	98	97	94	94	96	98	100	88	97
G..A	91	96	98	97	96	98	96	96	72	98	92	93	98	77	99	98
G..G	89	91	93	93	95	94	93	97	98	98	93	97	99	94	99	98

SUPPLEMENTARY FIGURES

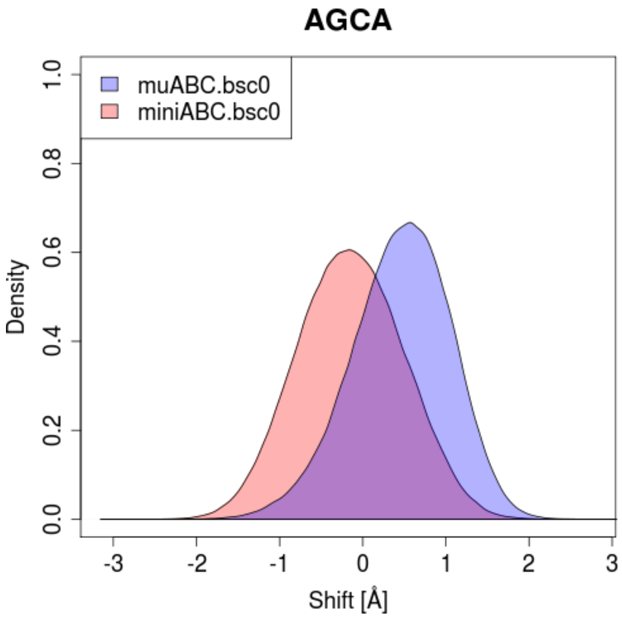

Figure S1. Shift distribution of the AGCA tetranucleotide obtained from μABC$_{BSC0}$-K and miniABC$_{BSC0}$-K. Both are bell-shaped Gaussian distributions, with a similar standard deviation, but different mean. All 1,631 pairs of other analogous marginal distributions were more similar one to the other.

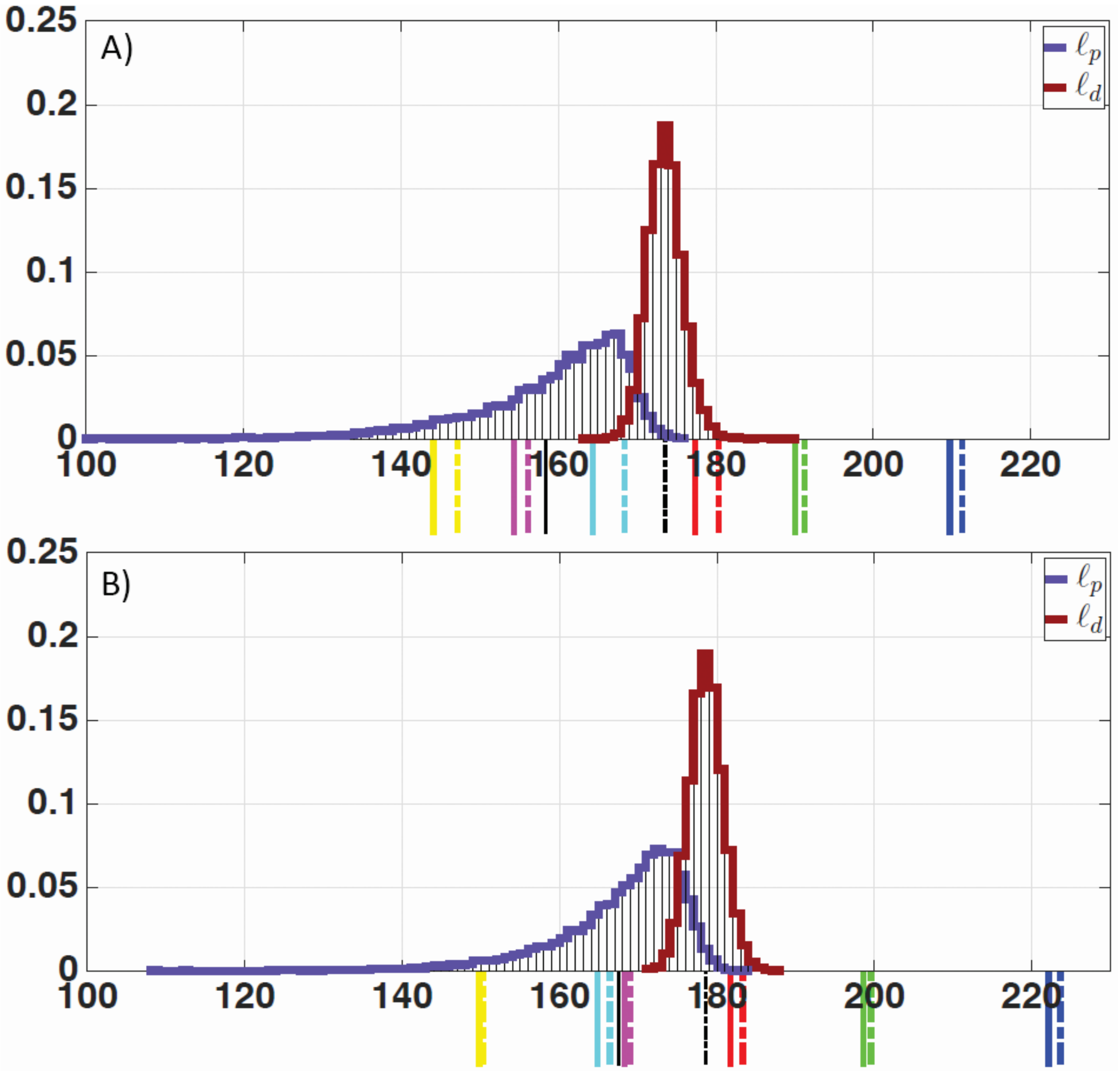

Figure S2. Spectra of ℓ_p (dark blue) and ℓ_d (dark red) persistence lengths computed over an ensemble of 10K sequences for A) PARMBSC0, and B) PARMBSC1 parameter sets, with mean for ℓ_p (black solid line) and mean for ℓ_d (black dashed line). The ℓ_p (coloured solid line) and ℓ_d (dashed solid line) values for the 6 distinct dinucleotide tandem repeats are also indicated in each case. The x-axis is in units of basepair, while the frequency is reported on the y-axis. Note that using an average rise of 0.33 nm, the peaks reported between 160 to 180 base pairs represent persistence lenths between 52 to 59 nm.

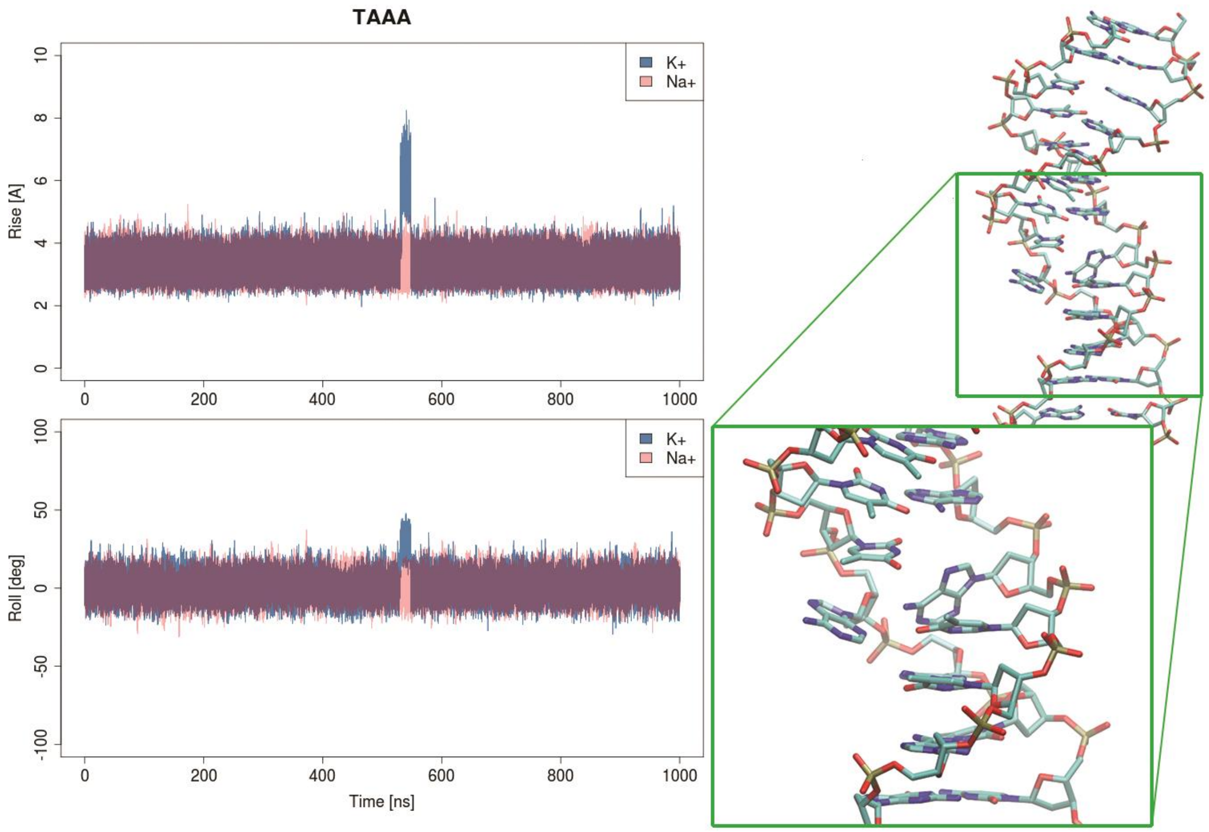

Figure S3. Time evolution of rise and roll for the TAAA tetranucleotide. The trajectory performed in K^+ (blue) shows the formation of a reversible kink near 550 ns, not present using Na^+ (pink). During the formation of the kink, up to two consecutive adenines lose their Watson-Crick H-bonds and are partially un-stacked. Note that this local distortion does not affect the main double helical structure of the oligomer.

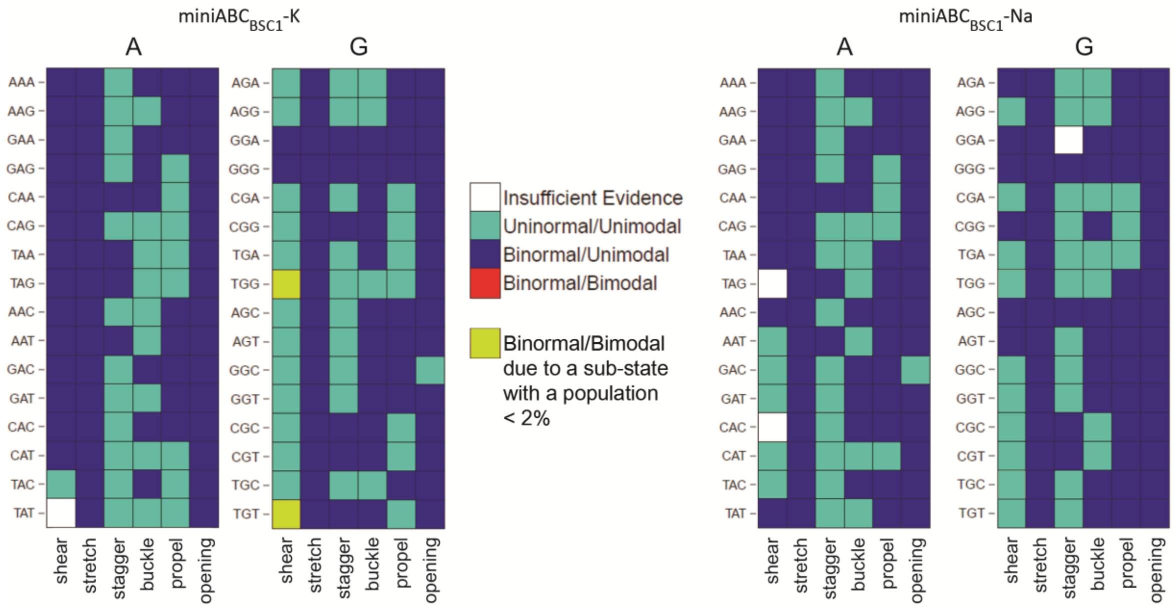

Figure S4. Structural polymorphisms (normality and modality) in intra-basepair helical conformations for all distinct trinucleotides. Results obtained from miniABC$_{BSC1}$-K and miniABC$_{BSC1}$-Na.

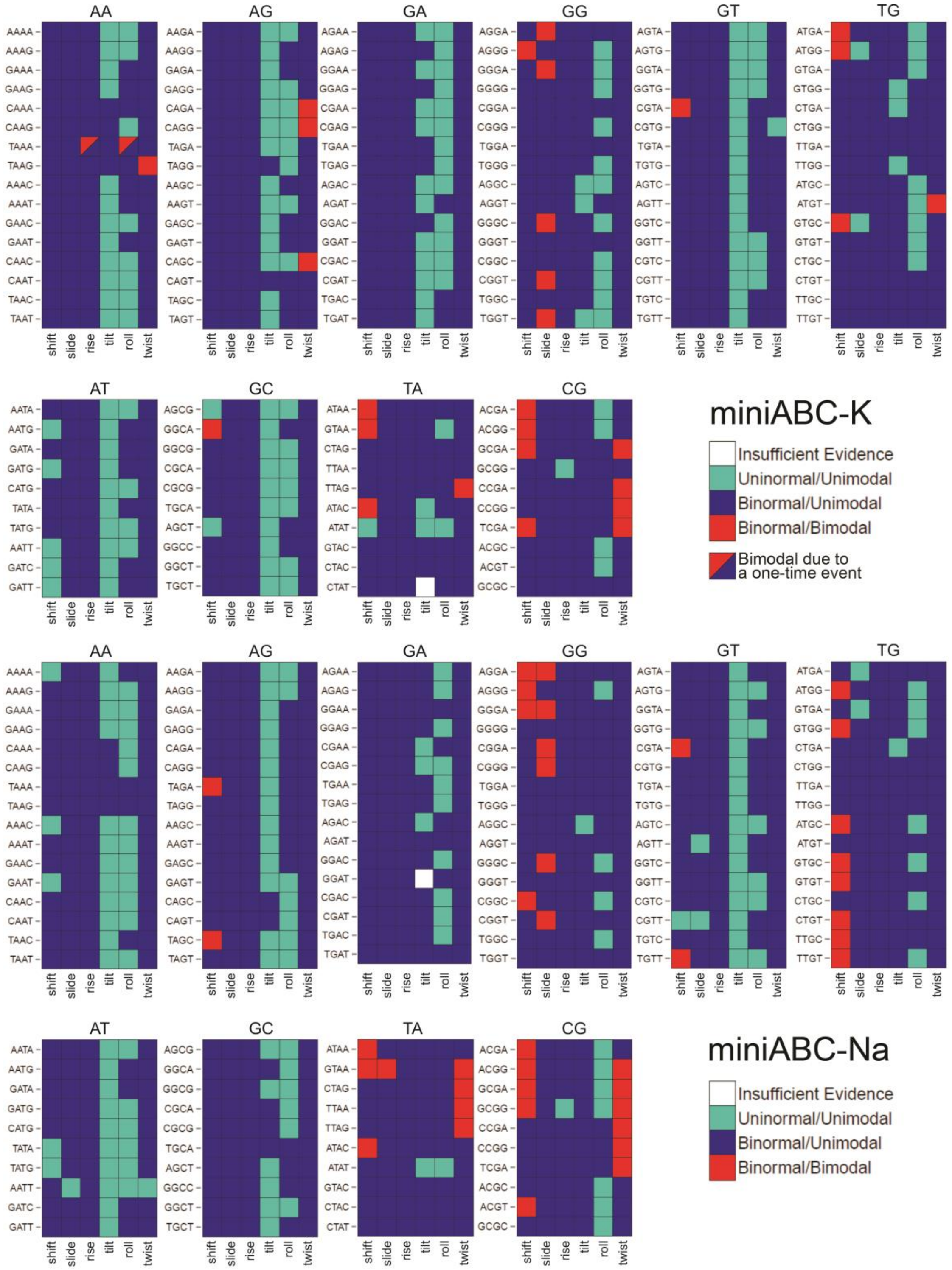

Figure S5. Structural polymorphisms (normality and modality) in inter-basepair helical conformations for all the 136 distinct tetranucleotides. Results obtained from miniABC$_{BSC1}$-K (top) and miniABC$_{BSC1}$-Na (bottom). Tetranucleotides classified as binormal/bimodal (red) are considered as polymorphic (exist in two clear conformational sub-states).

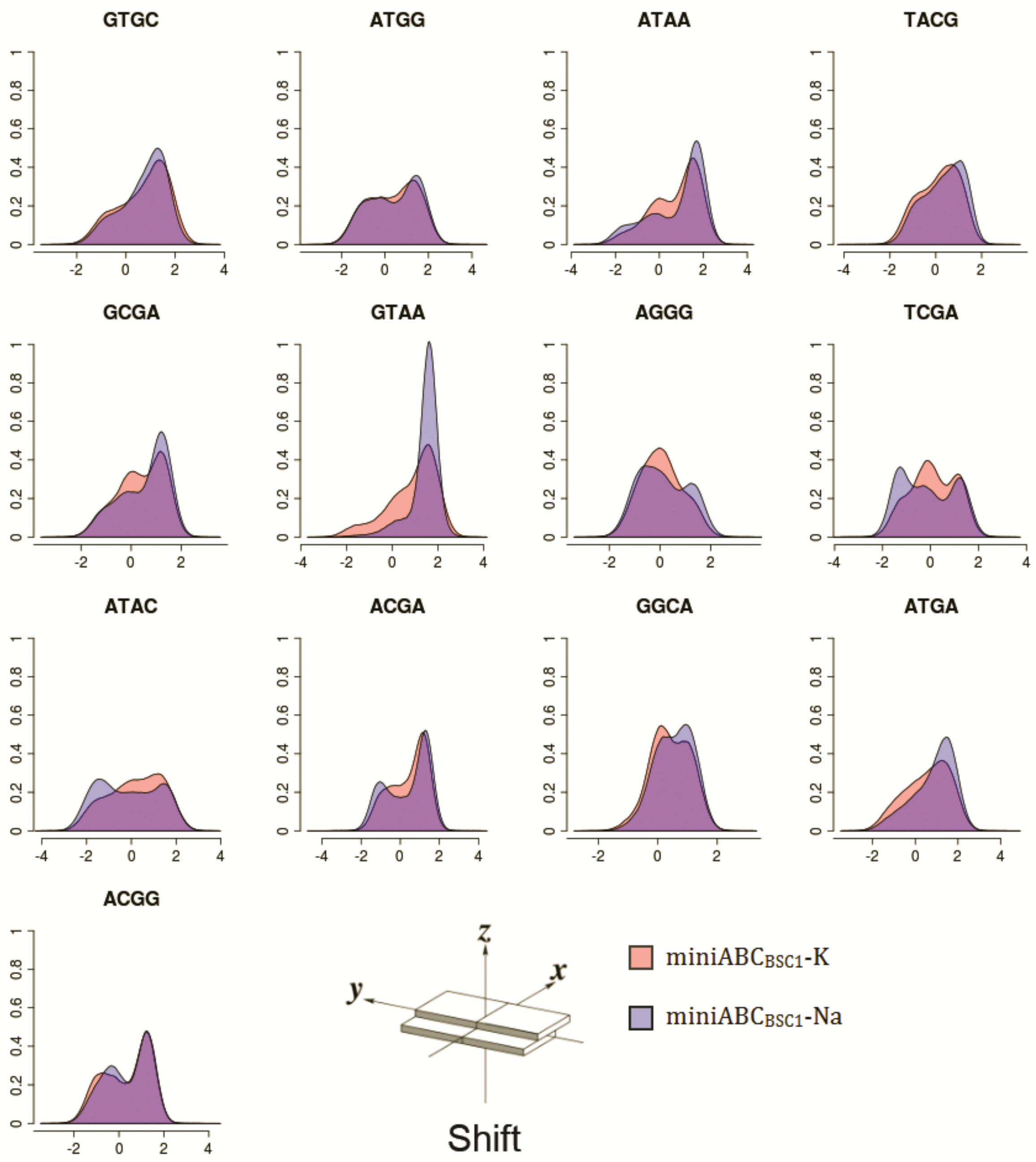

Figure S6. Normalized shift distributions for all the bimodal cases found in the miniABC$_{BSC1}$-K dataset, overlapped with their counterpart computed using Na+. X-axes represent the shift helical parameter in Å.

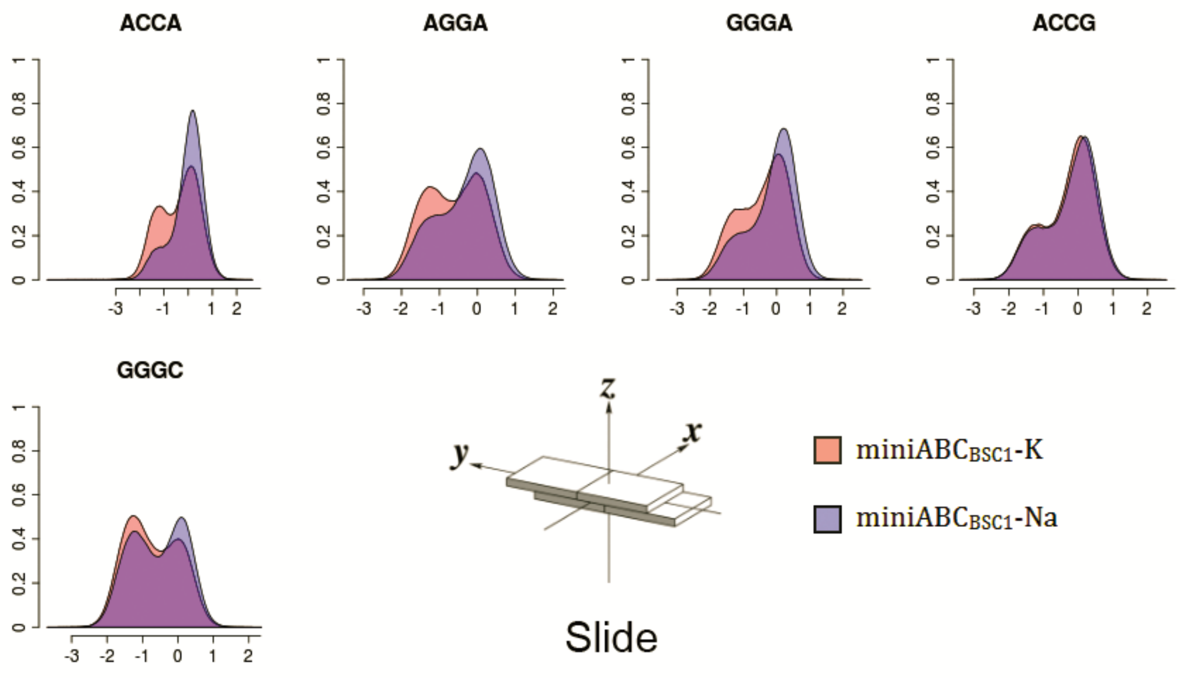

Figure S7. Normalized slide distributions for all the bimodal cases found in the miniABC$_{BSC1}$-K dataset, overlapped with their counterpart computed using Na+. X-axes represent the slide helical parameter in Å.

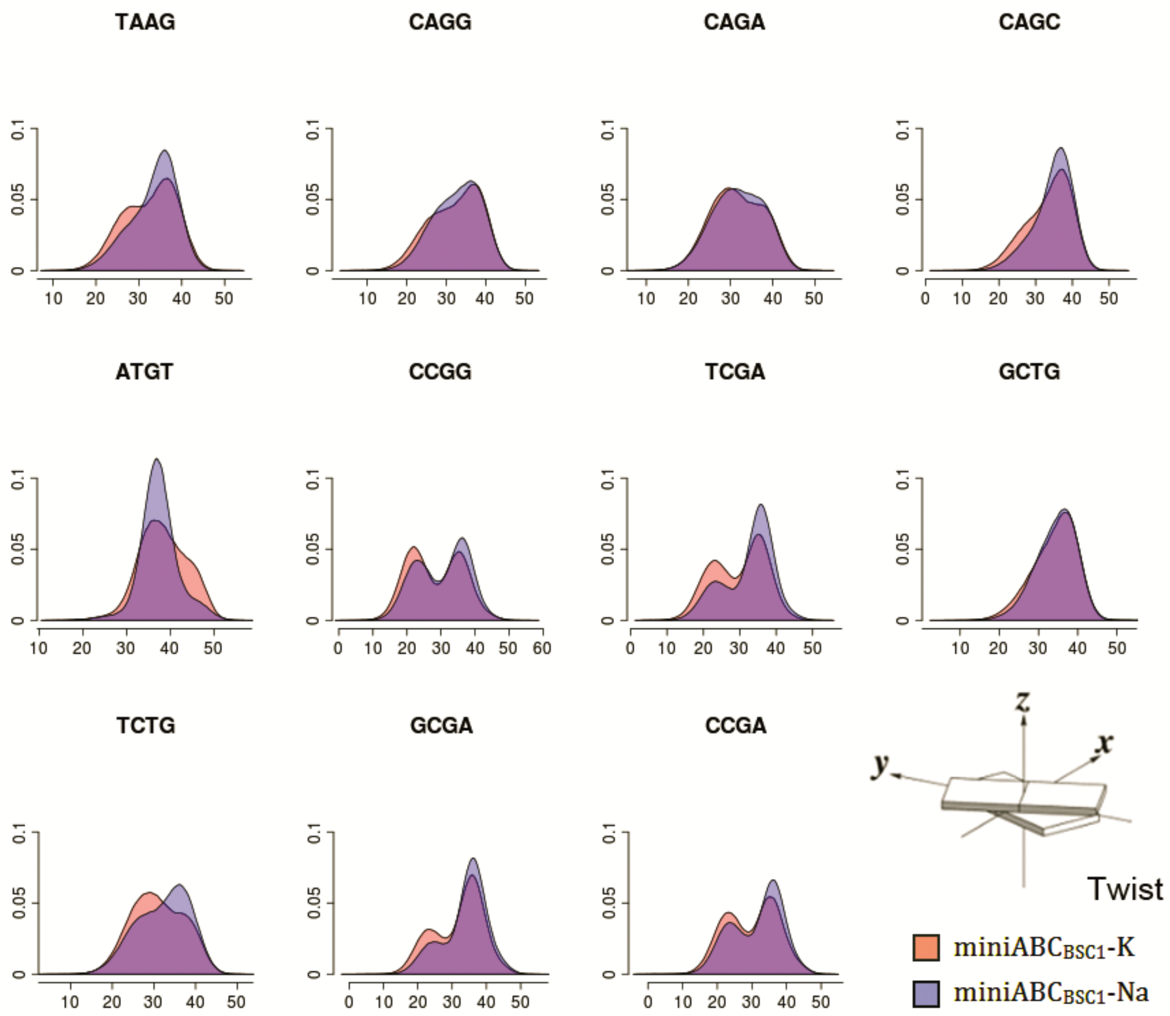

Figure S8. Normalized twist distributions for all the bimodal cases found in the miniABC$_{BSC1}$-K dataset, overlapped with their counterpart computed using Na+. The x-axes represent the twist helical parameter in degrees. Nota that the two peaks observed are in agreement with X-ray structures of DNA and protein-DNA complexes deposited in the Protein Data Bank(3).

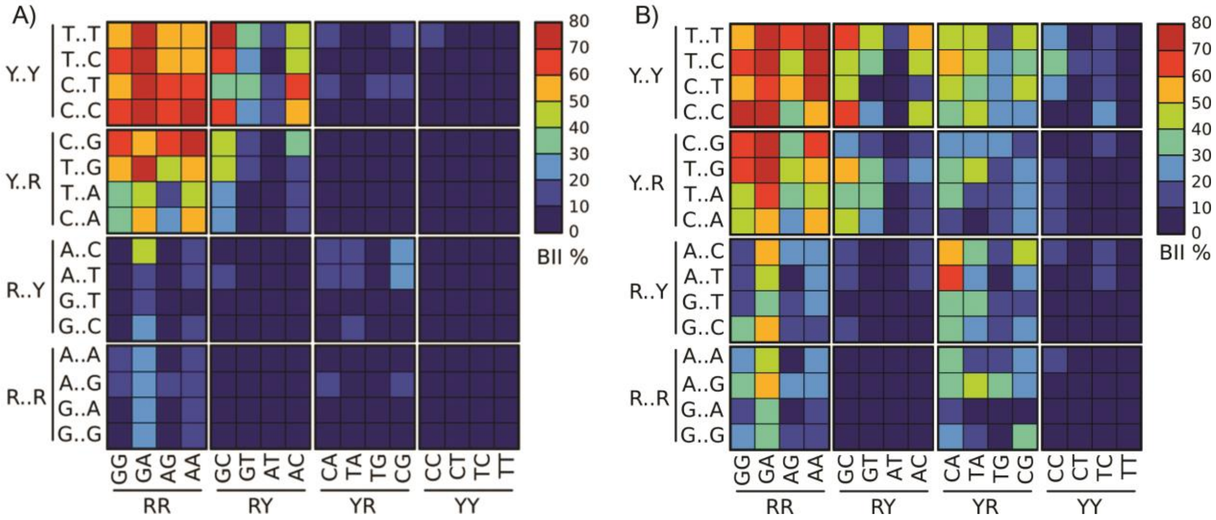

Figure S9. Sequence dependence of BII backbone conformations. The percentage occurrence of BII backbone states for the phosphodiester junction at the central base step of each of the 256 possible tetranucleotide sequences is shown (BII%), using the color code defined on the right (0% is dark blue; 80% is dark red). The sequences are arranged so that each column represents one of 16 dinucleotide steps, and each row corresponds to one of the 16 possible flanking sequences; columns and rows are further grouped on the basis of base type (R = purine and Y = pyrimidine). A) μABC$_{BSC0}$-K BII percentages(8); B) miniABC$_{BSC1}$-K BII percentages.

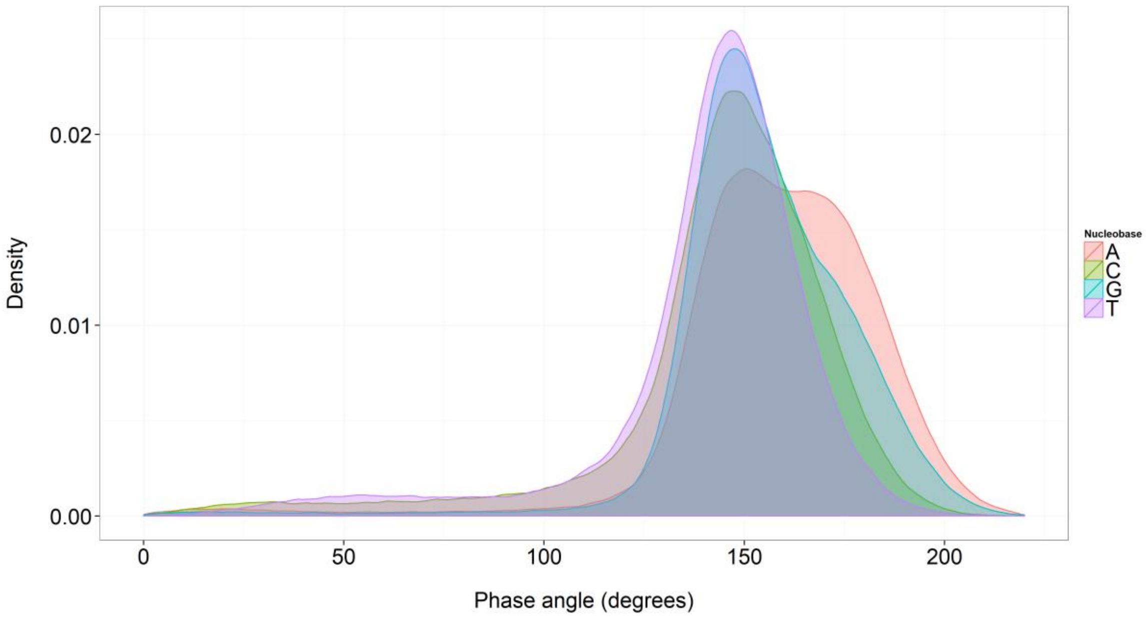

Figure S10. Normalized distribution of the P angle for A, C, G and T bases (in degrees), obtained from miniABC$_{BSC1}$-K dataset.

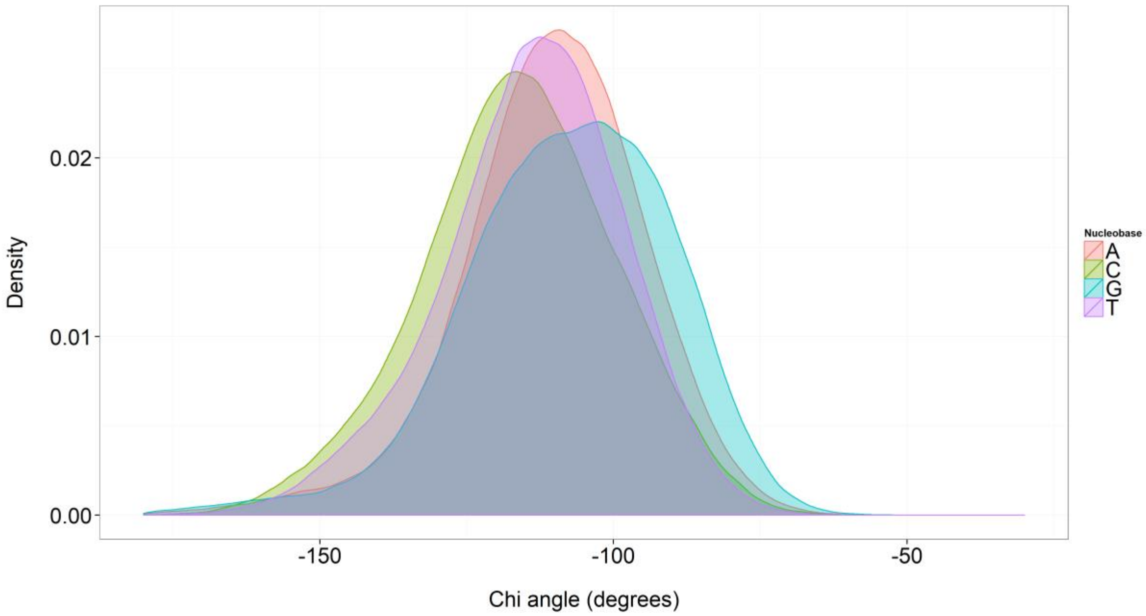

Figure S11. Normalized distribution of the χ angle for A, C, G and T bases (in degrees), obtained from miniABC$_{BSC1}$-K dataset.

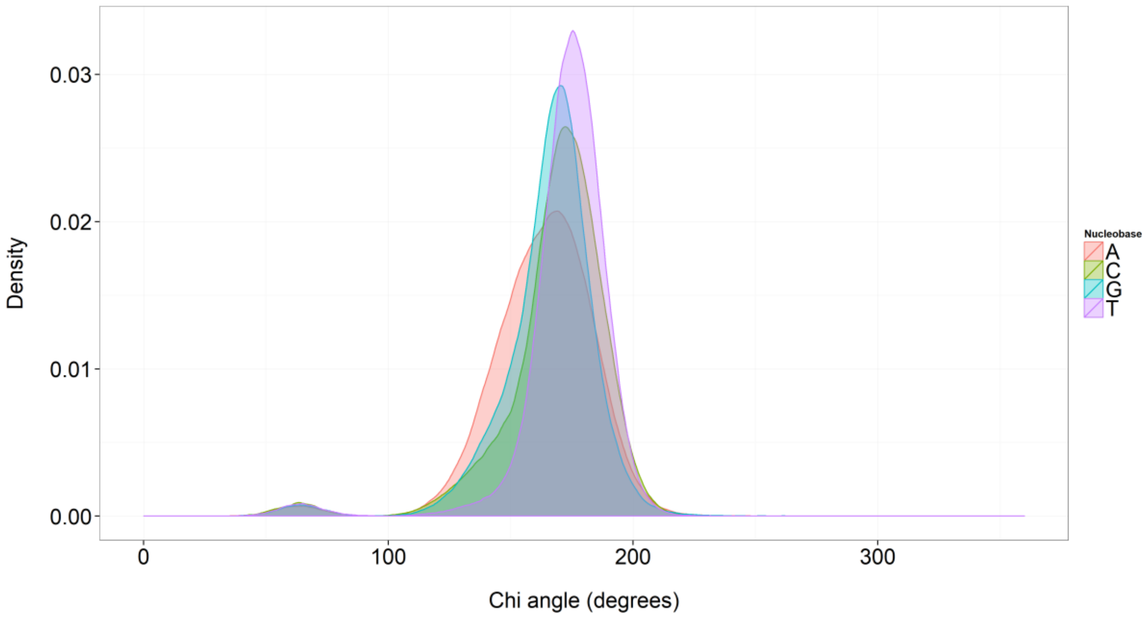

Figure S12. Normalized distribution of the β angle for A, C, G and T bases (in degrees), obtained from miniABC$_{BSC1}$-K dataset.

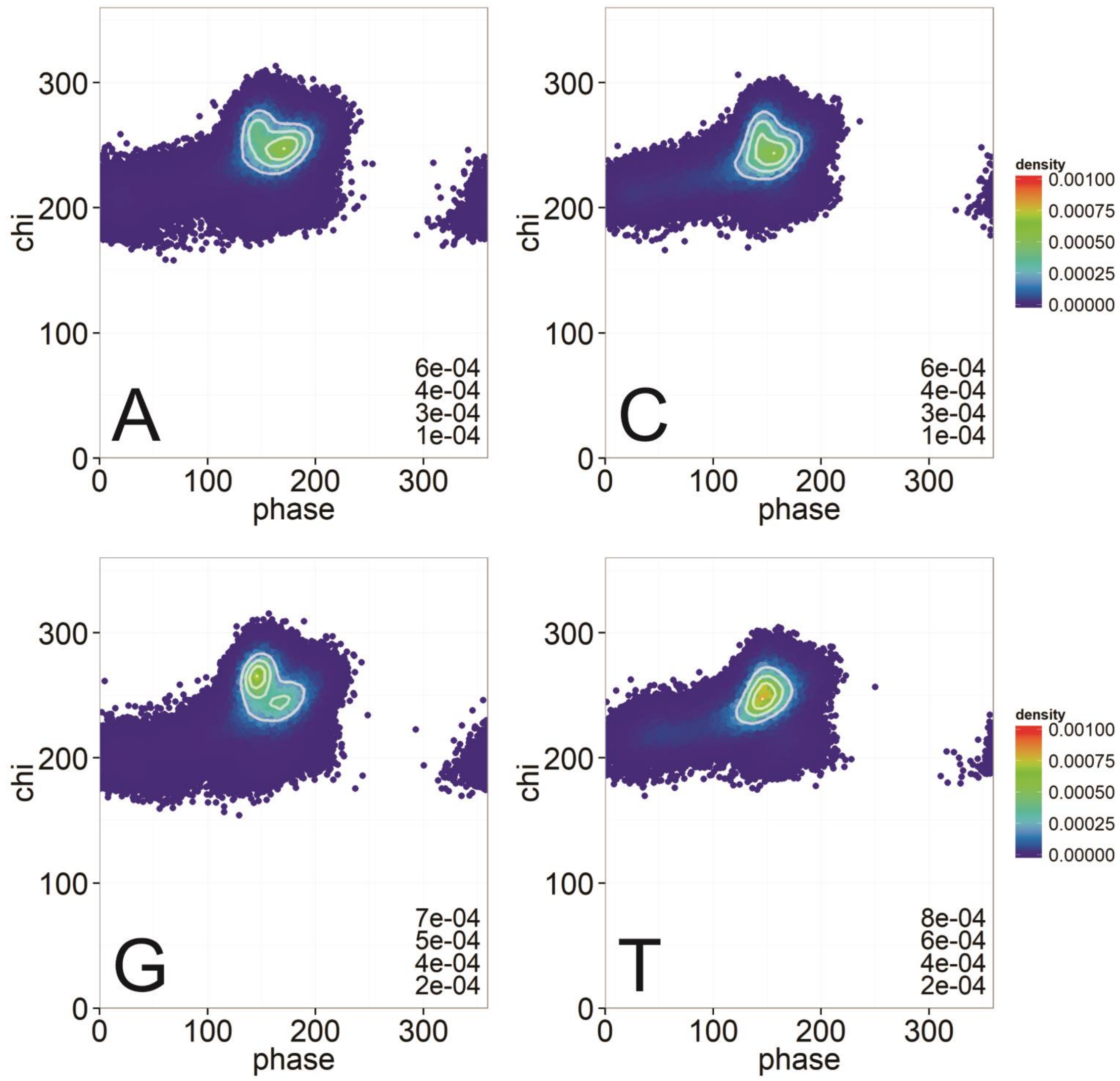

Figure S13. Sugar pseudorotation angle (phase) *vs* χ distribution plot (in degrees) obtained from miniABC$_{BSC1}$-K dataset for A, C, G and T bases.

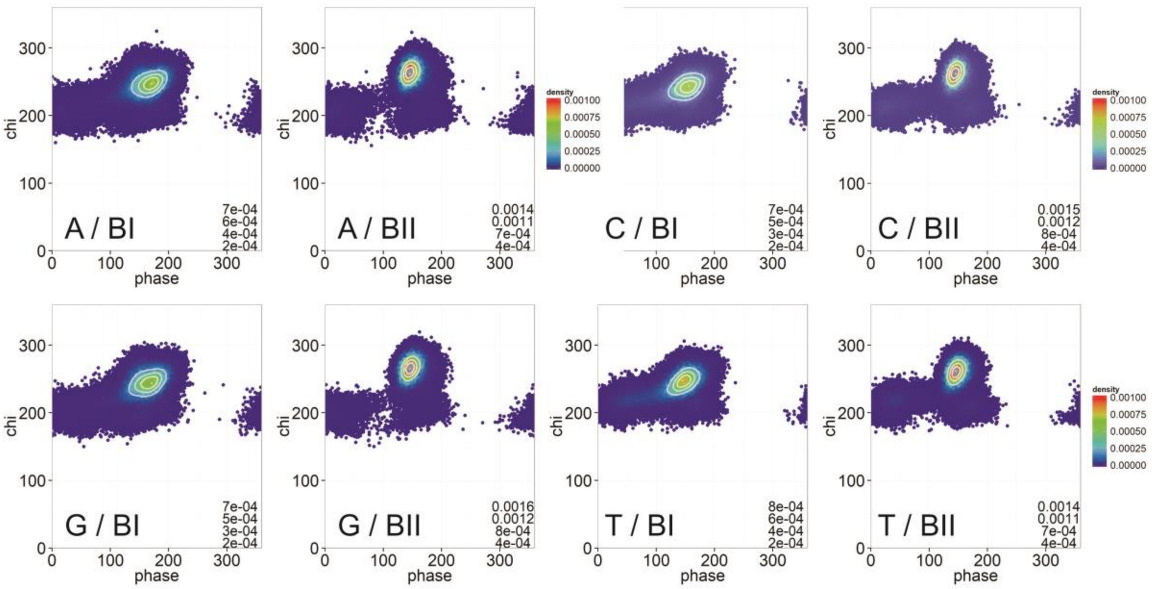

Figure S14. Sugar pseudorotation angle (phase) *vs* χ distribution plot (in degrees) obtained from miniABC$_{BSC1}$-K dataset and filtered according to BI/BII for A, C, G and T bases.

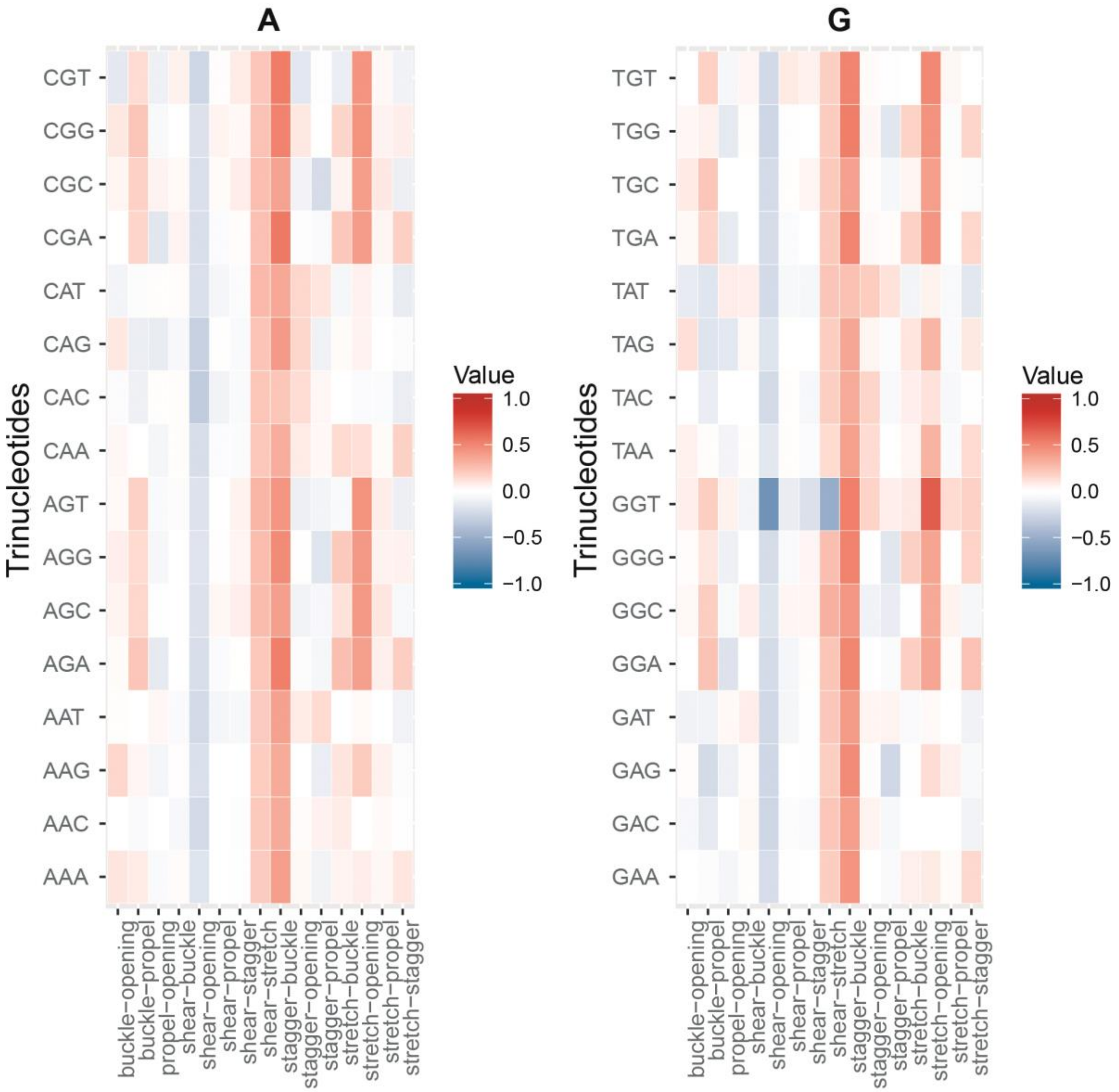

Figure S15. Correlation coefficients between intra-basepair helical parameters (shear, stretch, stagger, propeller, buckle and opening) belonging to the same base pair in the Watson strand. Results obtained from miniABC$_{BSC1}$-K dataset for all bps.

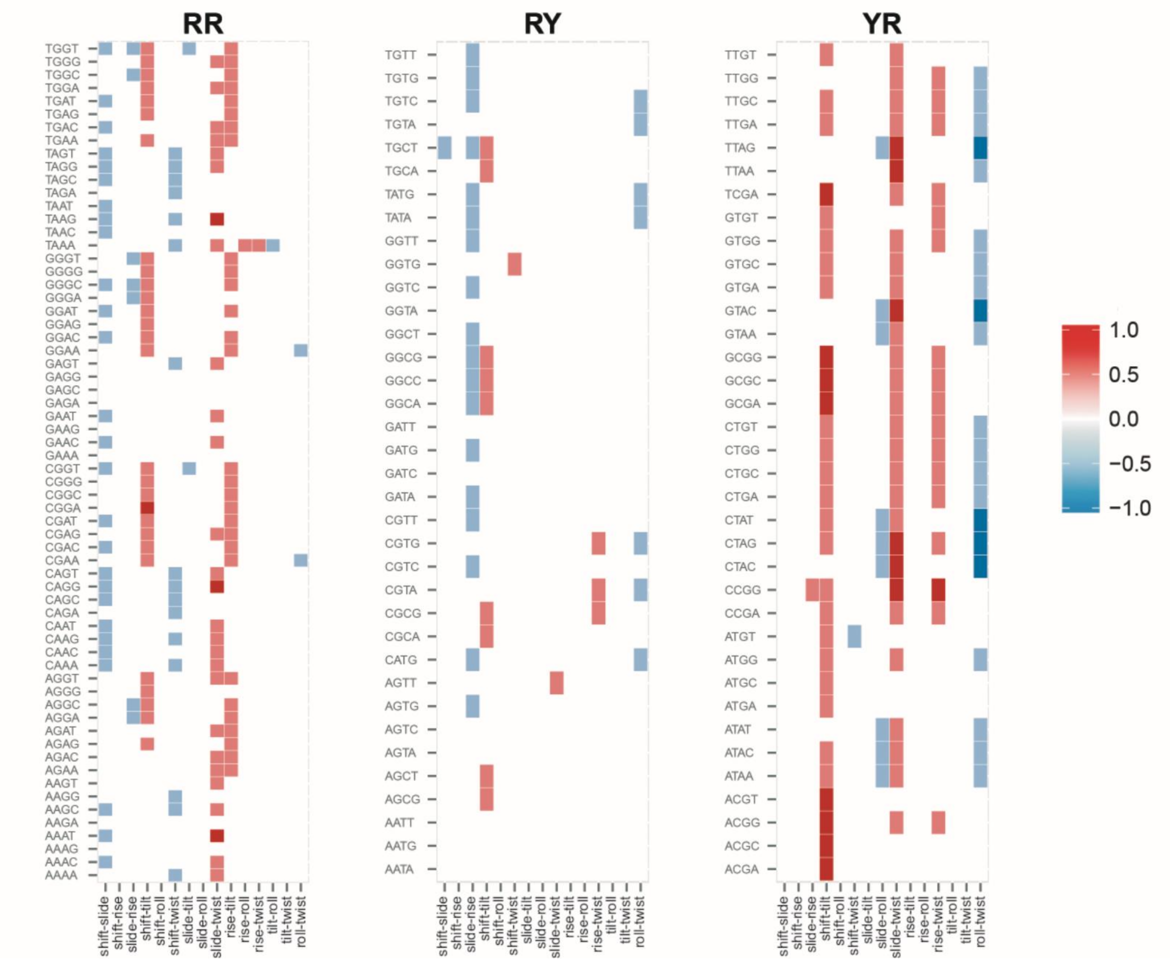

Figure S16. Correlation coefficients between inter-basepair helical parameters (shift, slide, rise, tilt, roll, and twist) belonging to the same step in the Watson strand. Results obtained from miniABC$_{BSC1}$-K dataset for all RR, RY and YR bps.

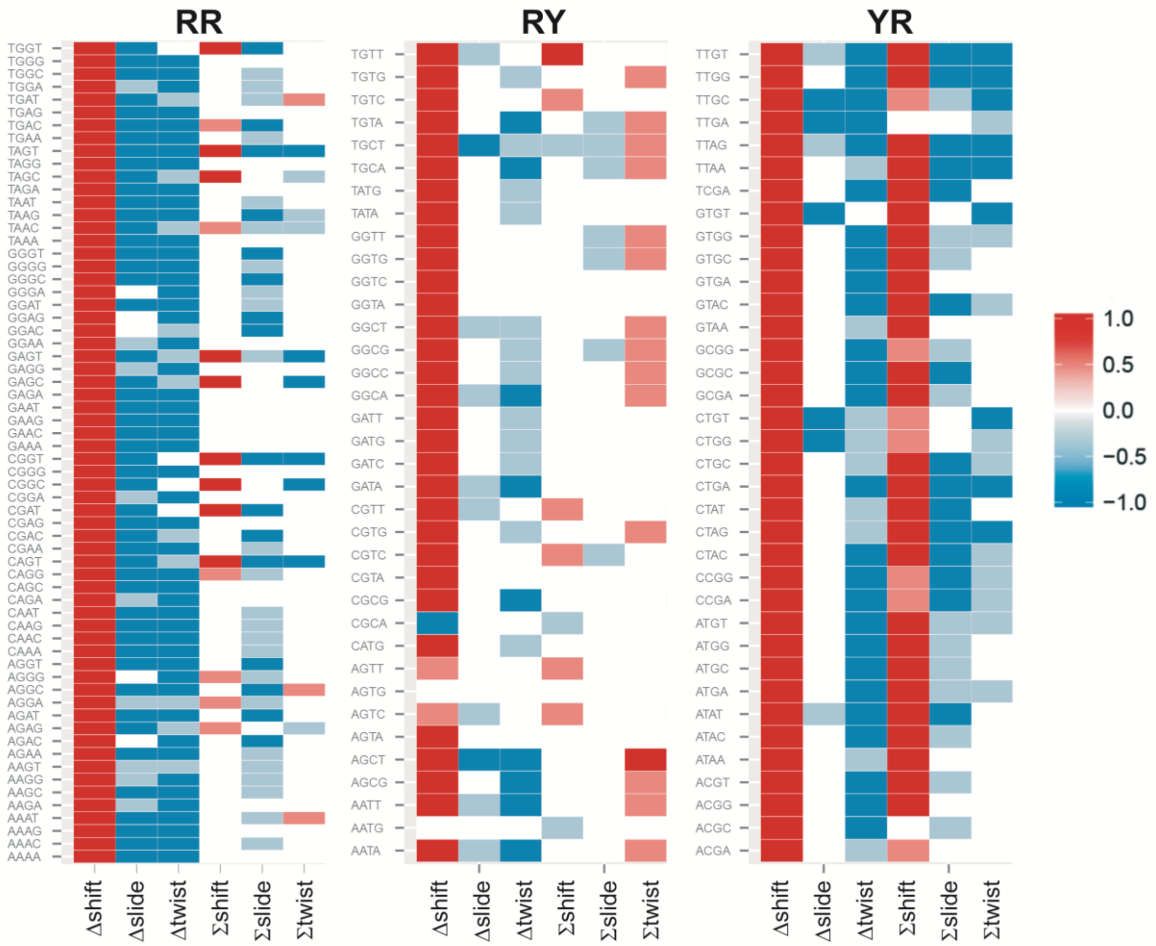

Figure S17. Correlation coefficients between differences (Δ) and sums (Σ) of inter-basepair parameters and the BII state in the central junction. Results obtained from miniABC$_{BSC1}$-K dataset for all steps grouped by RR, RY and YR.

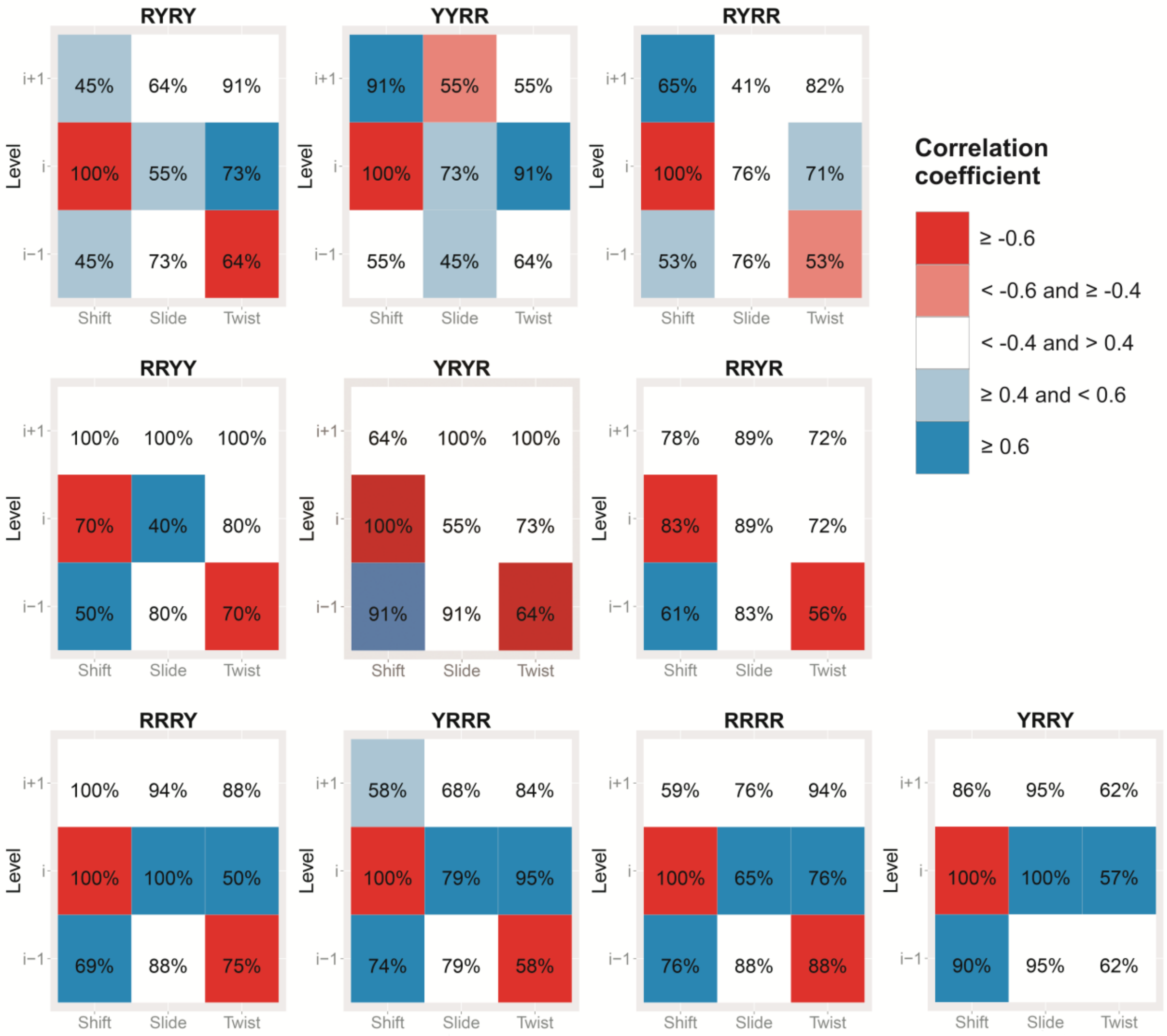

Figure S18. Correlation coefficients between shift, slide, or twist at the positions i-1 (5'-side), i, and i+1 (3'-side), and the backbone substate at the junction of inter-basepair i in the Watson strand. Results obtained from miniABC$_{BSC1}$-K dataset. The numbers inside each cell represent the % of specific tetranucleotides within a given family that give rise to the correlation.

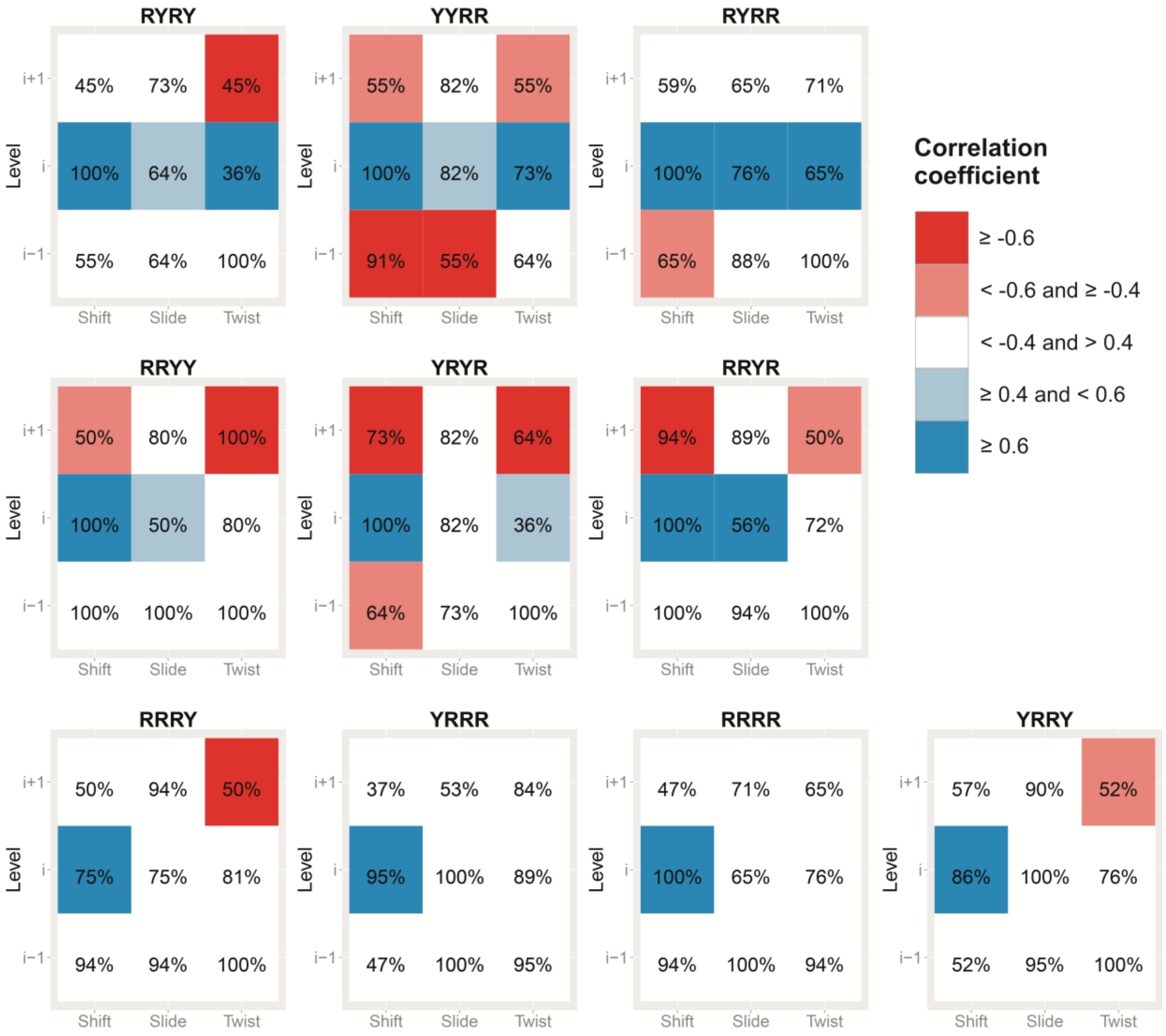

Figure S19. Correlation coefficients between shift, slide, or twist at the positions i-1 (5'-side), i, and i+1 (3'-side), and the backbone substate at the junction of inter-basepair i in the Crick strand. Note that we refer everything to the Watson strands (see Methods), so in this plot, RRRR means YYYY since we are analyzing the correlation with the Crick strand. Results obtained from miniABC$_{BSC1}$-K dataset.

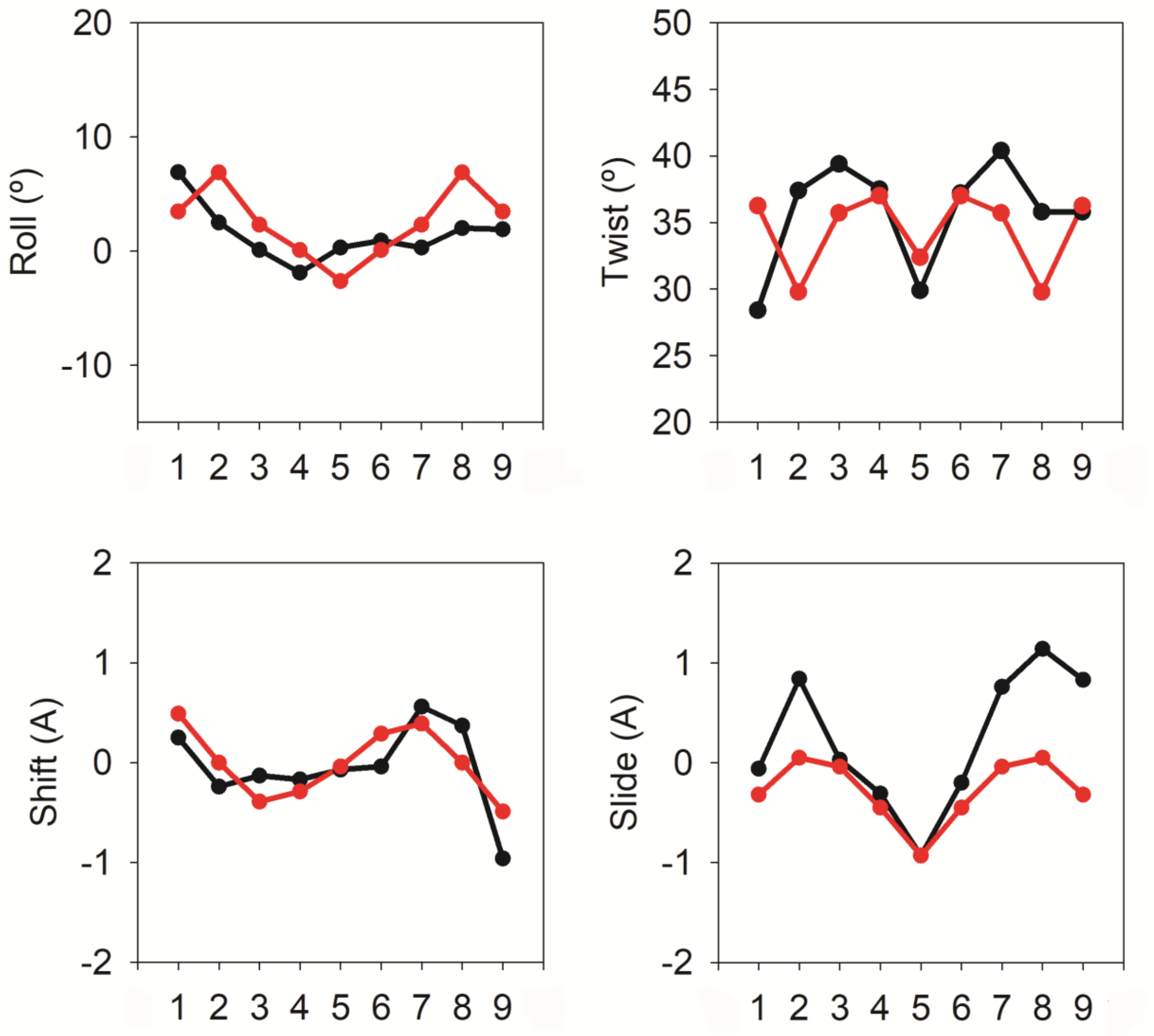

Figure S20. Comparison between the X-ray experimental structure with PDB id 1ILC (resolution 2.2 Å) and the conformation predicted by the miniABC$_{BSC1}$-K dataset. Four intra-basepair parameters were predicted (red lines), and compared with experiment (black lines). The x-axis label represent the basepair step number in the 5’→3’ without considering the capping bps.

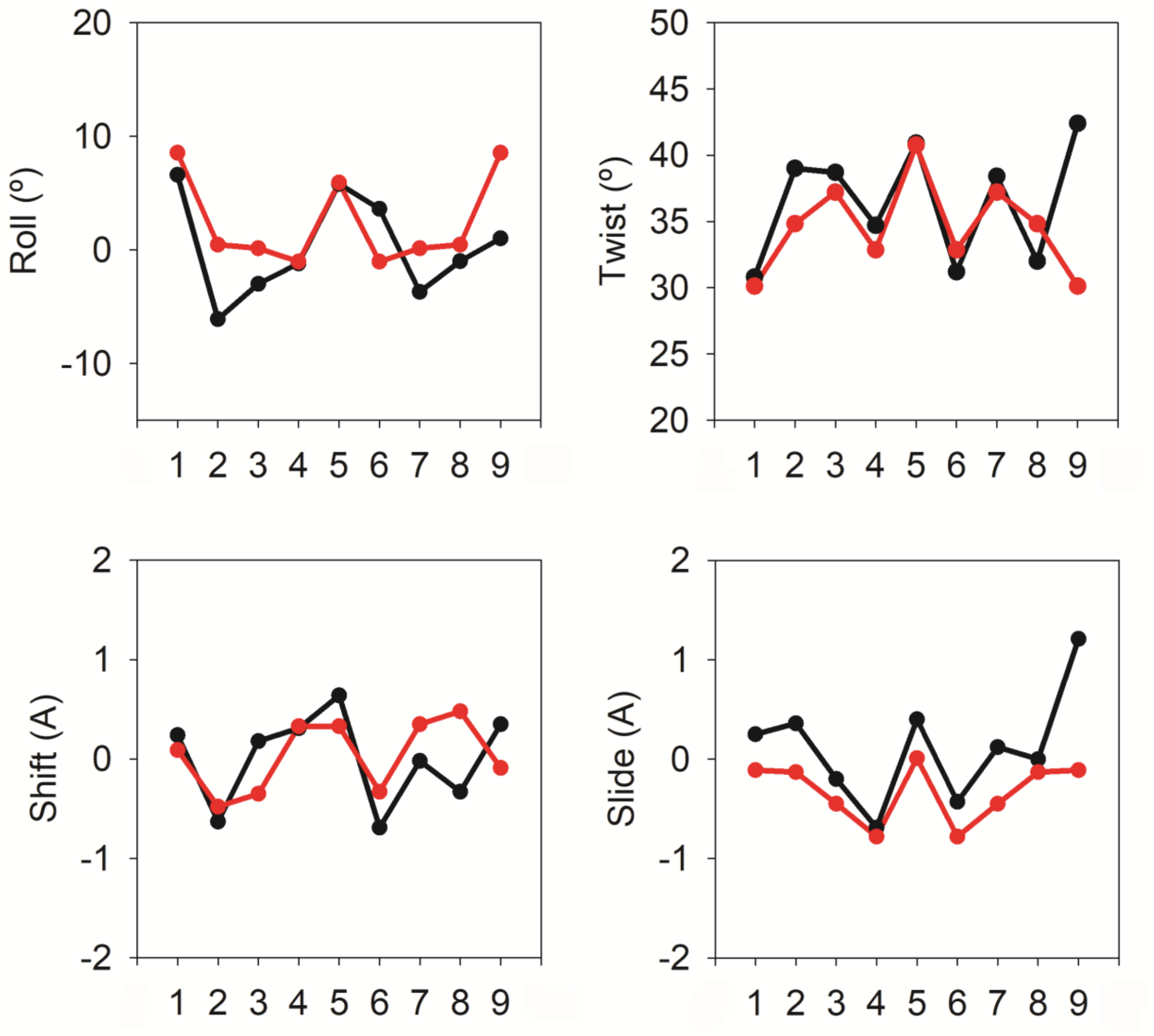

Figure S21. Comparison between the X-ray experimental structure with PDB id 1HQ7 (resolution 2.1 Å) and the conformation predicted by the miniABC$_{BSC1}$-K dataset. Four intra-basepair parameters were predicted (red lines), and compared with experiment (black lines). The x-axis label represent the basepair step number in the 5'→3' without considering the capping bps.

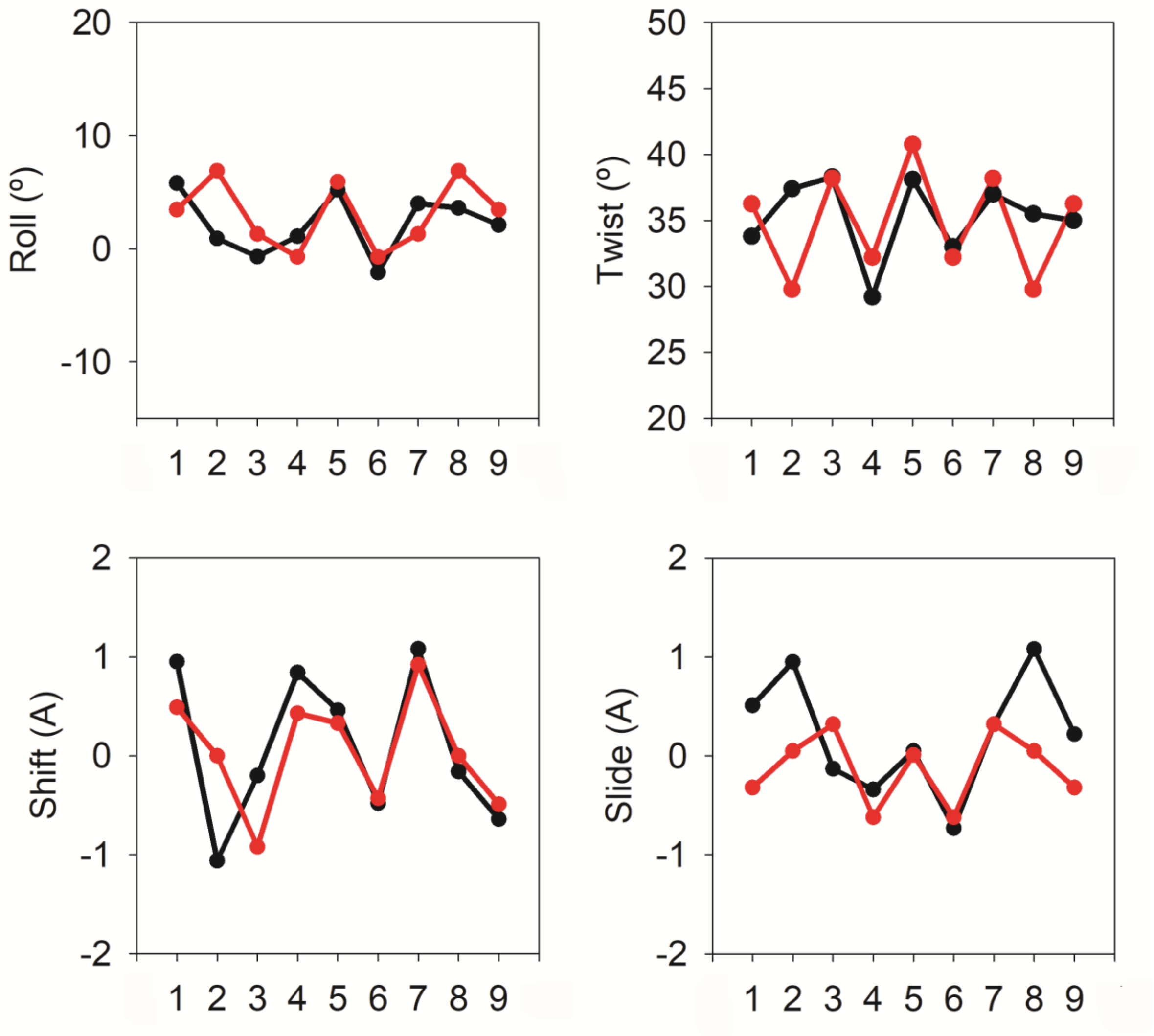

Figure S22. Comparison between the X-ray experimental structure with PDB id 424D (resolution 2.7 Å) and the conformation predicted by the miniABC$_{BSC1}$-K dataset. Four intra-basepair parameters were predicted (red lines), and compared with experiment (black lines). The x-axis label represent the basepair step number in the 5'→3' without considering the capping bps.

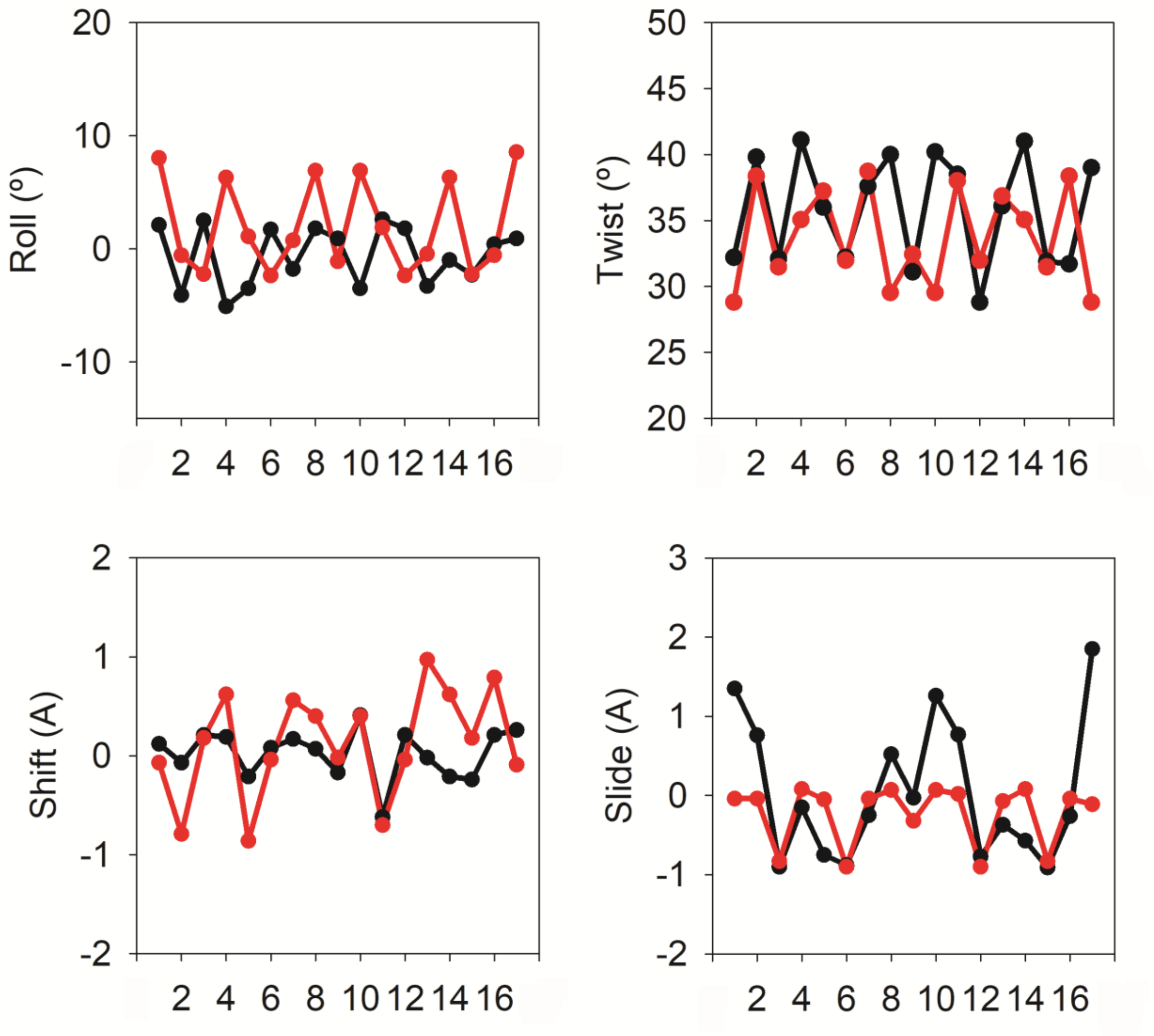

Figure S23. Comparison between the X-ray experimental structure with PDB id 5F9I (resolution 3.0 Å) and the conformation predicted by the miniABC$_{BSC1}$-K dataset. Four intra-basepair parameters were predicted (red lines), and compared with experiment (black lines). The x-axis label represent the basepair step number in the 5'→3' without considering the capping bps.

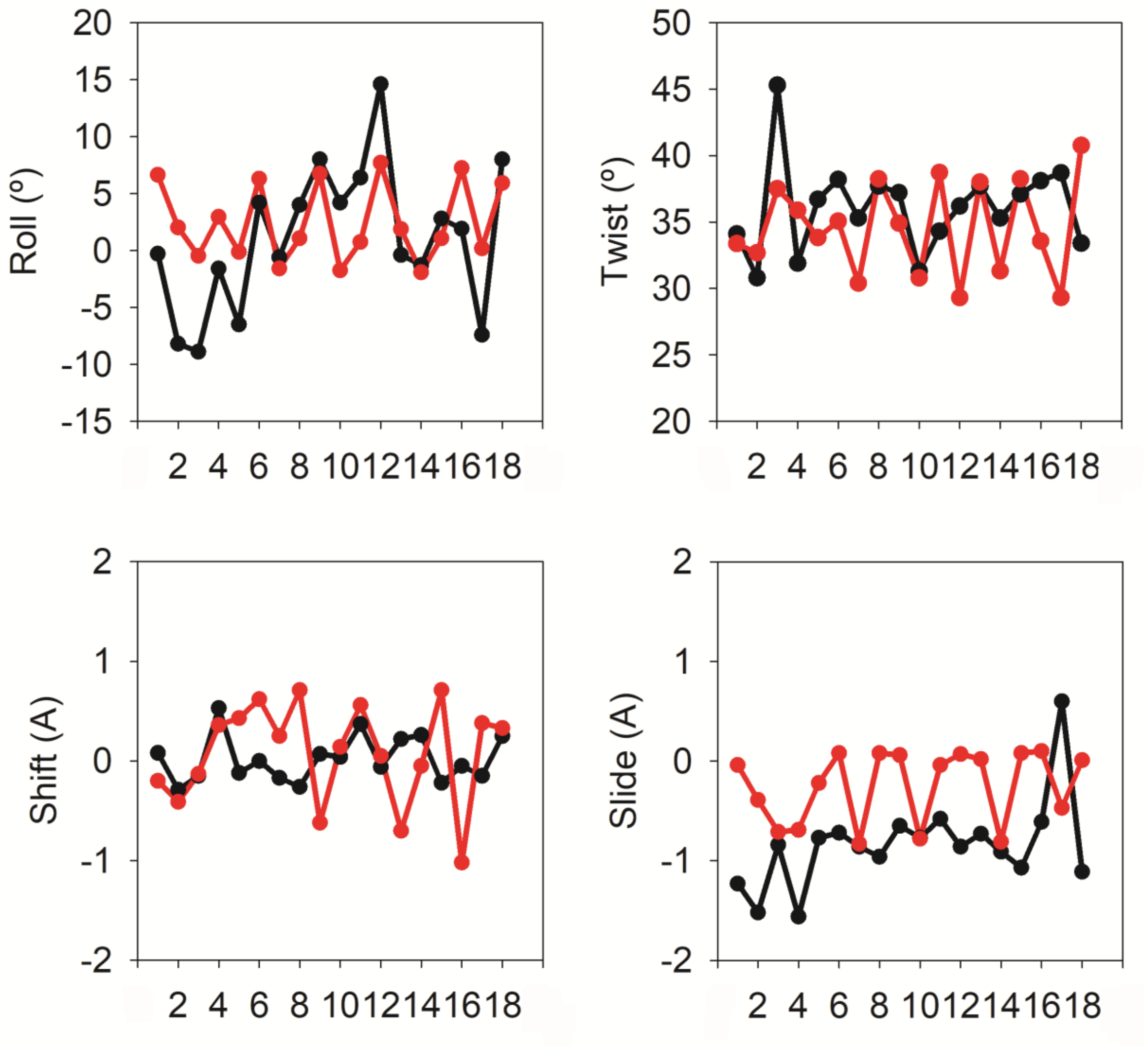

Figure S24. Comparison between the solution NMR experimental structure with PDB id 2JYK and the conformation predicted by the miniABC$_{BSC1}$-K dataset. Four intra-basepair parameters were predicted (red lines), and compared with experiment (black lines). The x-axis label represent the basepair step number in the 5'→3' without considering the capping bps.

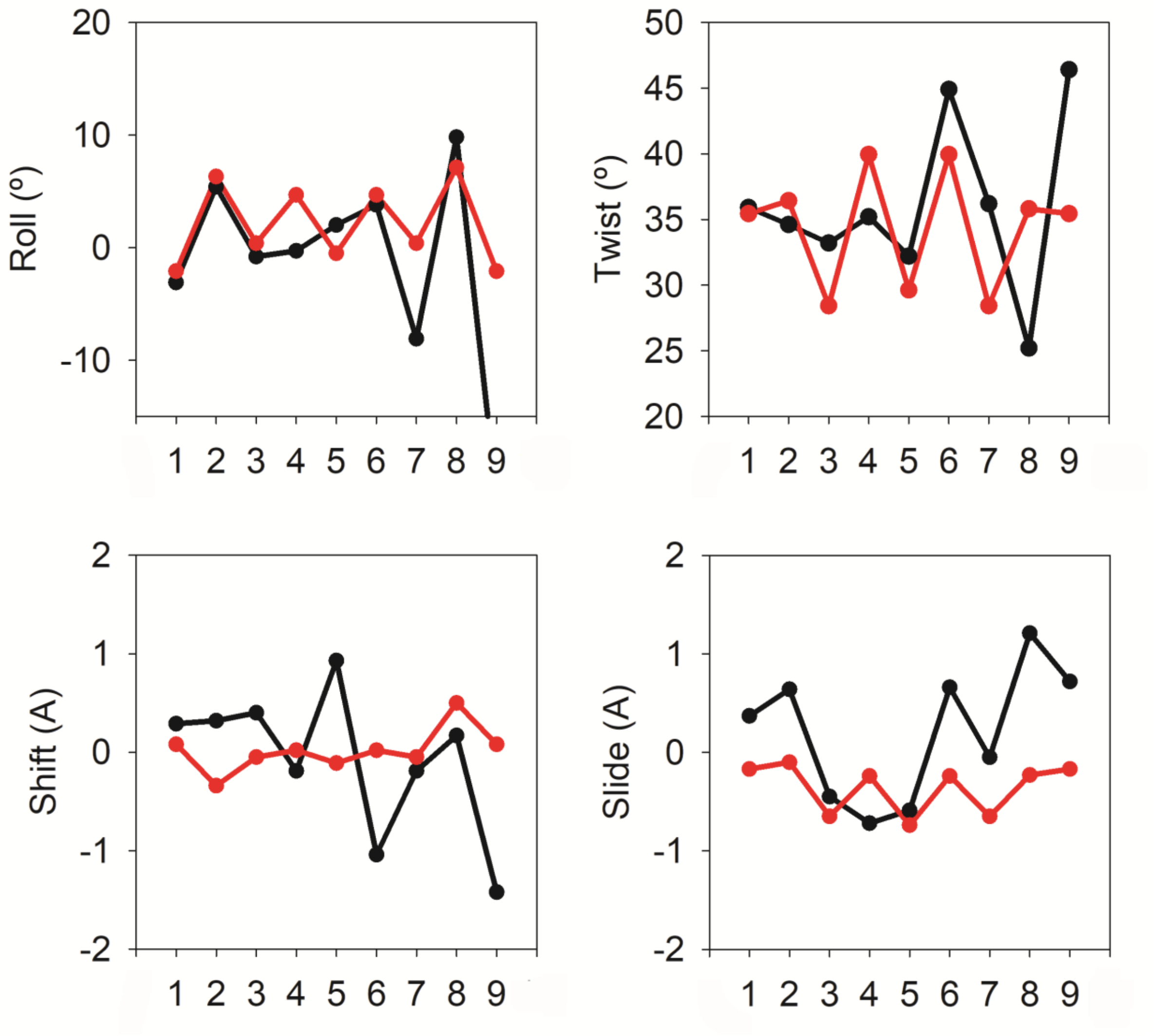

Figure S25. Comparison between the X-ray experimental structure with PDB id 1DN9 (resolution 2.2 Å) and the conformation predicted by the miniABC$_{BSC1}$-K dataset. Four intra-basepair parameters were predicted (red lines), and compared with experiment (black lines). The x-axis label represent the basepair step number in the 5'→3' without considering the capping bps.

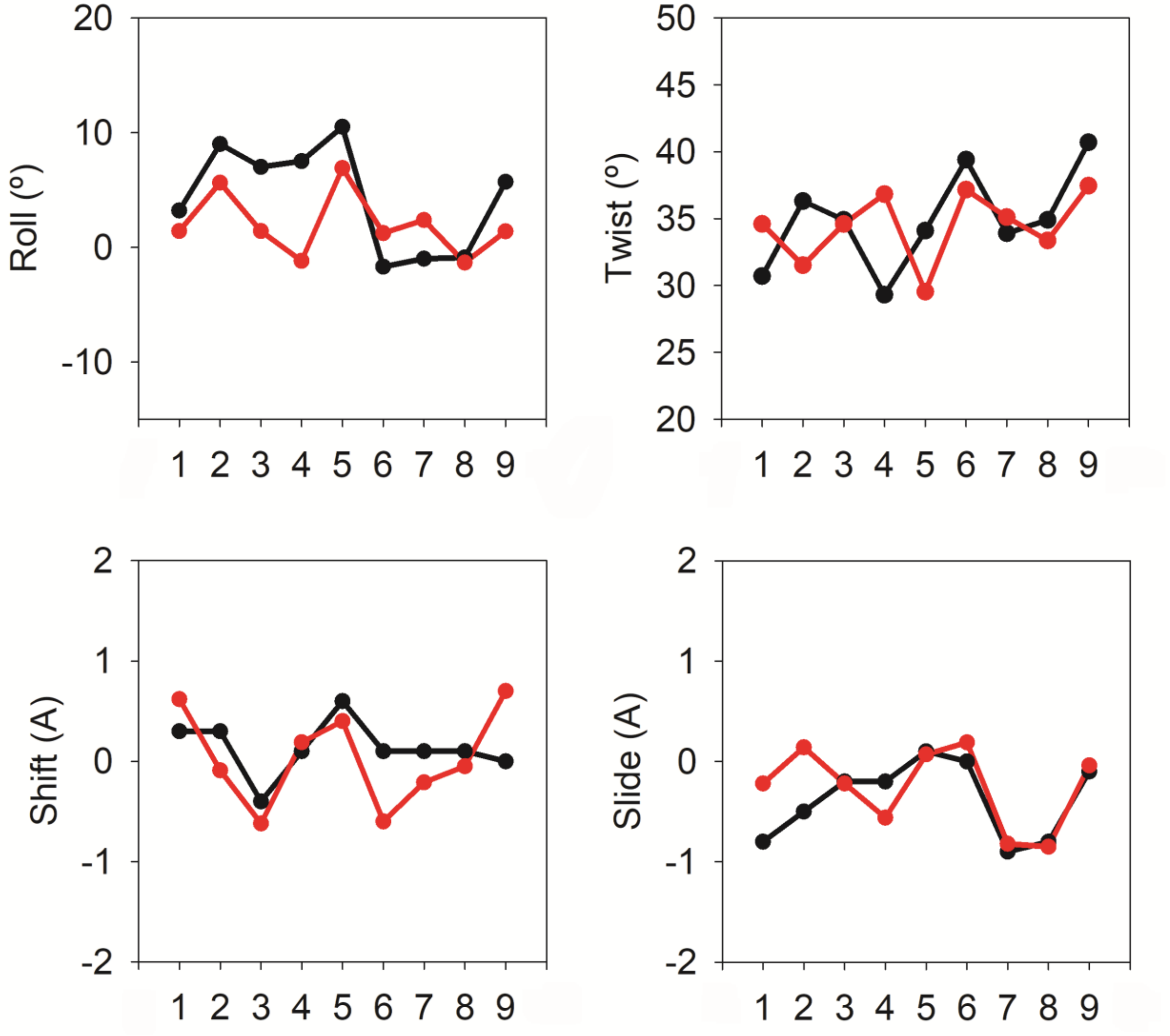

Figure S26. Comparison between the NMR experimental structure with sequence d(GpCpTpApGpCpGpApGpTpCpC) determined previously(9) and the conformation predicted by the miniABC$_{BSC1}$-K dataset. Four intra-basepair parameters were predicted (red lines), and compared with experiment (black lines). The x-axis label represent the basepair step number in the 5'→3' without considering the capping bps. Note that these graphics were done using the data in Suppl. Tables S10 and S11 of Dans *et al*. work(9), and the miniABC webserver https://mmb.irbbarcelona.org/miniABC/.

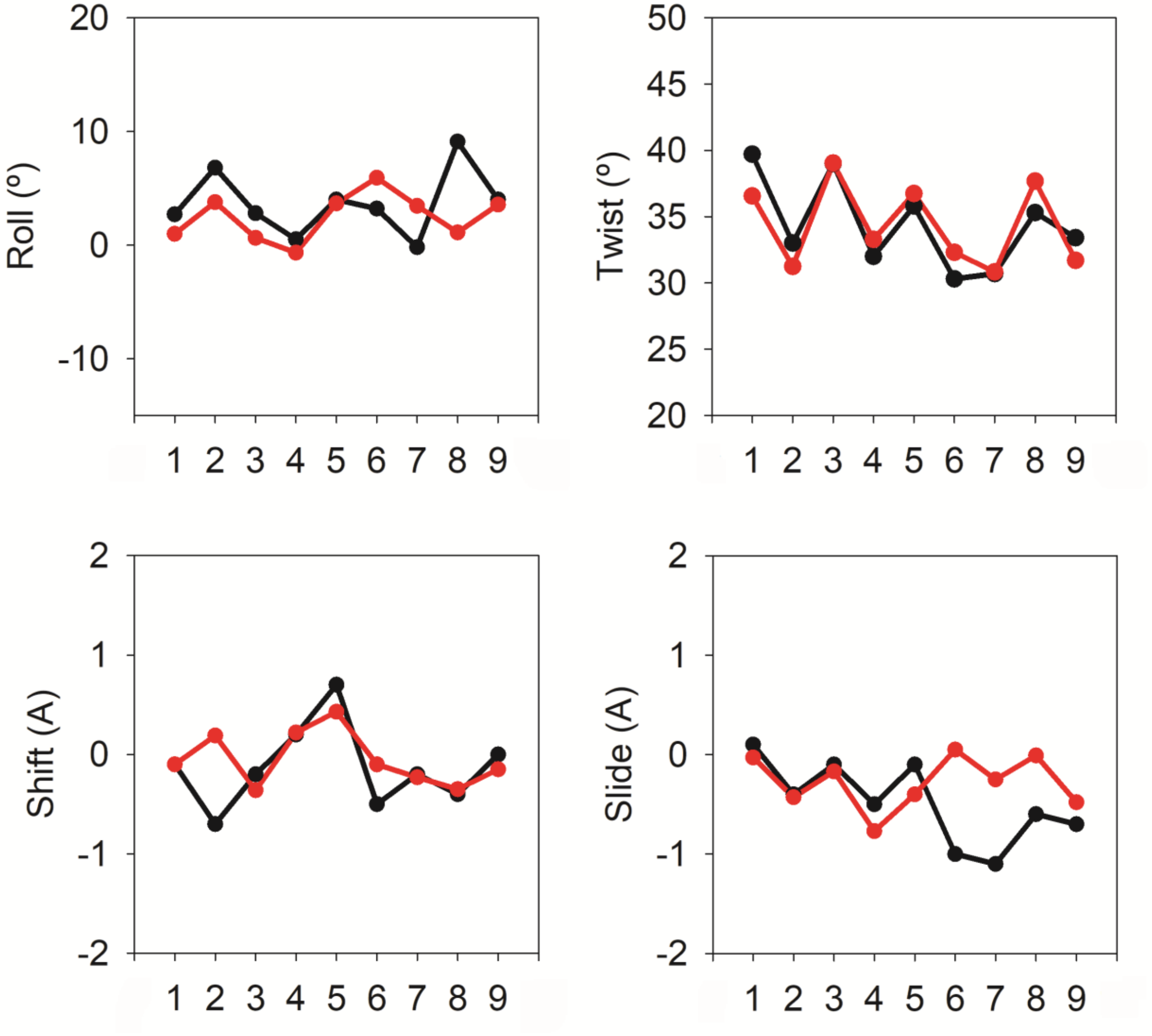

Figure S27. Comparison between the NMR experimental structure with sequence d(GpGpApGpApCpCpApGpApGpG) determined previously(9) and the conformation predicted by using the miniABC$_{BSC1}$-K dataset. Four intra-basepair parameters were predicted (red lines), and compared with experiment (black lines). The x-axis label represent the basepair step number in the 5'→3' without considering the capping bps Note that these graphics were done using the data in Suppl. Tables S12 and S13 of Dans *et al*. work(9), and the miniABC webserver https://mmb.irbbarcelona.org/miniABC/.

REFERENCES

1. Schwarz,G. (1978) Estimating the Dimension of a Model. *Ann. Stat.*, **6**, 461–464.
2. Kass,R.E. and Raftery,A.E. (1995) Bayes Factors. *J. Am. Stat. Assoc.*, **90**, 773–795.
3. Dans,P.D., Pérez,A., Faustino,I., Lavery,R. and Orozco,M. (2012) Exploring polymorphisms in B-DNA helical conformations. *Nucleic Acids Res.*, **40**, 10668–78.
4. de Helguero,F. (1904) Sui Massimi Delle Curve Dimorfiche. *Biometrika*, **3**, 84.
5. Schilling,M.F., Watkins,A.E. and Watkins,W. (2002) Is Human Height Bimodal? *Am. Stat.*, **56**, 223–229.
6. Lindley,D. V. (1959) Information Theory and Statistics. *Solomon Kullback* . New York: John Wiley and Sons, Inc.; London: Chapman and Hall, Ltd.; 1959. Pp. xvii, 395. $12.50. *J. Am. Stat. Assoc.*, **54**, 825–827.
7. Mitchell,J.S., Glowacki,J., Grandchamp,A.E., Manning,R.S. and Maddocks,J.H. (2017) Sequence-Dependent Persistence Lengths of DNA. *J. Chem. Theory Comput.*, **13**, 1539–1555.
8. Balaceanu,A., Pasi,M., Dans,P.D., Hospital,A., Lavery,R. and Orozco,M. (2017) The Role of Unconventional Hydrogen Bonds in Determining BII Propensities in B-DNA. *J. Phys. Chem. Lett.*, **8**, 21–28.
9. Dans,P.D., Ivani,I., Hospital,A., Portella,G., González,C. and Orozco,M. (2017) How accurate are accurate force-fields for B-DNA? *Nucleic Acids Res.*, **45**, 4217–4230.
